# Characteristics and birth outcomes of pregnant adolescents compared to older women: An analysis of individual level data from 140,000 mothers from 20 RCTs

**DOI:** 10.1016/j.eclinm.2022.101309

**Published:** 2022-02-26

**Authors:** Nadia Akseer, Emily Catherine Keats, Pravheen Thurairajah, Simon Cousens, Ana Pilar Bétran, Brietta M. Oaks, David Osrin, Ellen Piwoz, Exnevia Gomo, Faruk Ahmed, Henrik Friis, José Belizán, Kathryn Dewey, Keith West, Lieven Huybregts, Lingxia Zeng, Michael J. Dibley, Noel Zagre, Parul Christian, Patrick Wilfried Kolsteren, Pernille Kaestel, Robert E. Black, Shams El Arifeen, Ulla Ashorn, Wafaie Fawzi, Zulfiqar Ahmed Bhutta, The Global Young Women's Nutrition Investigators Group

**Affiliations:** a Centre for Global Child Health, Hospital for Sick Children, Toronto, ON M5G 0A4, Canada; b London School of Hygiene and Tropical Medicine, London, United Kingdom; c Dalla Lana School of Public Health, University of Toronto, Toronto, Canada; d Institute for Global Health and Development, The Aga Khan University, Karachi, Pakistan; e World Health Organization, Switzerland; f University of Rhode Island, United States; g Institute for Global Health, University College London, United Kingdom; h The Bill and Melinda Gates Foundation, United States; i University of Zimbabwe, Zimbabwe; j Griffith University, Australia; k University of Copenhagen, Denmark; l UNC Chapel Hill, United States; m University of California, Davis, United States; n Johns Hopkins Bloomberg School of Public Health, United States; o International Food Policy Research Institute, United States; p School of Public Health, Xi'an Jiaotong University Health Science Centre, China; q The University of Sydney, Australia; r UNICEF Regional Office for West and Central Africa, Senegal; s Johns Hopkins Bloomberg School of Public Health, United States; t Ghent University, Belgium; u University of Copenhagen, Denmark; v Johns Hopkins Bloomberg School of Public Health, United States; w ICDDRB, Bangladesh; x Faculty of Medicine and Health Technology, Tampere University, Finland; y Harvard T.H. Chan School of Public Health, United States

**Keywords:** Adolescence, Age, Determinants, Pregnancy, Birth outcomes

## Abstract

**Background:**

Adolescence is a critical period of maturation when nutrient needs are high, especially among adolescents entering pregnancy. Using individual-level data from 140,000 participants, we examined socioeconomic, nutrition, and pregnancy and birth outcomes for adolescent mothers (10–19 years) compared to older mothers in low and middle-income countries.

**Methods:**

This study was conducted between March 16, 2018 and May 25, 2021. Data were obtained from 20 randomised controlled trials of micronutrient supplementation in pregnancy. Stratified analyses were conducted by age (10–14 years, 15–17 years, 18–19 years, 20–29 years, 30–39 years, 40+ years) and geographical region (Africa, Asia). Crude and confounder-adjusted means, prevalence and relative risks of pregnancy, nutrition and birth outcomes were estimated using multivariable linear and log-binomial regression models with 95% confidence intervals.

**Findings:**

Adolescent mothers comprised 31.6% of our data. Preterm birth, small-for-gestational age (SGA), low birthweight (LBW) and newborn mortality followed a U-shaped trend in which prevalence was highest among the youngest mothers (10–14 years) and then reduced gradually, but increased again for older mothers (40+ years). When compared to mothers aged 20–29 years, there was a 23% increased risk of preterm birth, a 60% increased risk of perinatal mortality, a 63% increased risk of neonatal mortality, a 28% increased risk of LBW, and a 22% increased risk of SGA among mothers 10–14 years. Mothers 40+ years experienced a 22% increased risk of preterm birth and a 103% increased risk of stillbirth when compared to the 20–29 year group.

**Interpretation:**

The youngest and oldest mothers suffer most from adverse pregnancy and birth outcomes. Policy and programming agendas should consider both biological and socioeconomic/environmental factors when targeting these populations.

**Funding:**

Bill and Melinda Gates Foundation (Grant No: OP1137750).

**Research in context**

**Evidence before this study**

We used the following search terms to examine the available evidence on risk factors and outcomes among pregnant adolescents in low-income and middle-income countries (LMICs): (adolescent (MeSH) or adolescen* or teen* or youth*) and (pregnancy (MeSH) or pregnan* or parity or gravid*) and (pregnancy outcome (MeSH) or risk factors (MeSH) or outcome* or risk* or complication*) and [LMIC filter provided by Cochrane]. The search was conducted on Nov 29, 2017, in Medline, and inclusion criteria included reviews published in 2000 or later on the topic. Synthesised results pointed to a complex interplay of factors that lead to adolescent pregnancies, including poverty, being out of school, being married, poor access to health services, and lacking in family and peer role models while associated outcomes included maternal mortality, preterm birth, low birthweight, neonatal mortality, and perinatal mortality.

**Added value of this study**

This study will add to the evidence base for understanding risk factors and outcomes associated with adolescent pregnancies. Given that most of the existing evidence is cross-sectional, with few prospective evaluations, one of the major advantages is the study's use of individual-level participant data (N∼140,000) from 20 micronutrient supplementation trials across Africa and Asia. In addition, we have disaggregated maternal age by 6 subgroups (10–14, 15–17, 18–19, 20–29, 30–39, and 40+ years) to better understand how risks and outcomes change across these sub-populations.

**Implications of all the available evidence**

This study highlights major differences in adverse outcomes across maternal age and supports previous research that found higher risks for adolescents and older mothers. It underscores the urgent need to prevent at-risk pregnancies and, where these pregnancies do occur, ensure that proper nutritional services, antenatal care, and care at birth are provided. This will be particularly important for the youngest mothers; and notably, for the oldest mothers (40+ years). Taken together, evidence from this study can be used to inform decisions and action among global agenda setters and policy makers, practitioners, and academic researchers in countries where adolescent and older mother pregnancy rates are high.

Alt-text: Unlabelled box

## Introduction

The cohort of adolescents living today is the largest in history, encompassing 1.2 billion girls and boys aged 10–19 years, the majority of whom live in a low-income or middle-income country (LMIC).^1^ Before their 20th birthday, 40% of girls in LMICs are married and almost 20% will have given birth.^2^ Among the youngest adolescents (<16 years), a staggering 2.5 million births occur in LMICs annually.^3^ The current SARS-CoV-2 pandemic has likely increased these estimates because of restrictions in health services, lack of contraceptive access, increased school dropouts, and a complex interplay of these factors with economic hardship and a deepening of gender-based disparities.^4^^,^^5^ Adolescent fertility is traditionally high in LMICs, particularly in Sub-Saharan Africa and Latin America, due to pervasive underlying factors such as poverty, poor access to health services, lack of education and employment opportunities, low female autonomy, cultural practices related to sexual health and marriage, and gender norms and roles.^2^^,^^6^

Adolescence is a critical period marking phenomenal changes including rapid physical, psychosocial, sexual and cognitive maturation, and nutrient needs of adolescents are higher than at any other stage in the lifecycle.^7^ Pregnant adolescent girls are a particularly vulnerable group since the demands of regular growth and development are augmented by the heightened nutritional requirements of supporting a fetus. It is, however, unclear whether pregnancy in adolescence will limit maternal growth, or whether girls with adequate nutrition will continue to grow on a normal trajectory.^8^, ^9^, ^10^ When there is competition for nutrients between the mother and fetus (i.e., the mother has inadequate nutrient intake and stores), studies have suggested a nutrition partitioning that favours the fetus.^10^, ^11^, ^12^

How age and underlying nutritional status affect pregnancy and birth outcomes is not yet fully understood. Analysis of survey data in Pakistan and Kenya^13^^,^^14^ suggests that, compared to older women of reproductive age, adolescents have poorer nutrition profiles. Obstructed labor (due to short stature and smaller pelvic size) was also found among young adolescents (10–14 years), though there were limited and conflicting data on birth and reproductive outcomes. An analysis of national surveys from 55 LMICs found that young mothers had a higher risk of poor health and mortality outcomes among their newborns than older mothers.^15^ A meta-analysis of 14 cohort studies conducted in LMICs found that nulliparous women aged <18 years had the highest risk of adverse birth and neonatal outcomes, including preterm birth, small-for-gestational age (SGA), neonatal and infant mortality, when compared to women 18–34 years with parity 1–2.^16^ However, other nutrition and reproductive health outcomes were not examined and data were not disaggregated for younger adolescent age groups. Though the proportion of births was extremely low, a prospective, multi-country study that disaggregated outcomes among pregnant girls <15 years and those 15–19 years found greater risks for preterm birth and low birthweight (LBW) among the youngest group.^17^

Given the high number of births occurring during adolescence (estimated at 20% of all births in some countries),^16^ understanding the relationship between maternal age and pregnancy and birth outcomes is important and could have major implications for policy and programming. This study aimed to empirically examine patterns in socioeconomic conditions, nutrition, and pregnancy and birth outcomes for adolescent mothers compared to older mothers in LMICs using a unique set of individual-level cohort data. Specific study objectives included: (i) describing baseline socioeconomic characteristics and the epidemiologic profile of key maternal and newborn health, nutrition and mortality outcomes by maternal age group (10–14 years, 15–17 years, 18–19 years, 20–29 years, 30–39 years, 40+ years) and geographic region (Asia versus Africa) and (ii) estimating covariate-adjusted age effects on specified outcomes (maternal anemia, preterm birth, stillbirth, perinatal mortality, neonatal mortality, LBW, SGA).

## Methods

### Data collection

The Global Young Women's Nutrition Investigators Group was established in 2016 as a voluntary global adolescent nutrition interest consortium aiming to study key health and nutritional outcomes in this population. As part of the consortium, we identified and collated individual participant data (IPD) from individually- and cluster-randomised trials of the effects of micronutrient supplementation interventions among pregnant girls and women (Table 1). A total of 20 trials with IPD for 140,000 mothers were obtained. The main analysis examined the effect of antenatal multiple micronutrient (MMN) supplementation on pregnant adolescents as compared to older mothers. This analysis is published separately and also details the systematic review process used to identify relevant randomised controlled trials (RCT), along with the process of establishing the collaboration.^18^ Eligibility criteria required trials to have been conducted in an LMIC and to have included at least 100 adolescents (10–19.9 years) in their sample. For the current study, we have included all 20 trials in pregnant girls and women for which we were able to obtain IPD; an acceptable approach with minimal bias even when pooling individual and cluster randomised trials.^19^ Pooling individual-level trial data that include adolescents is the best means of assessing health and birth outcomes within this population subset, given the absence of trials that recruit only pregnant adolescents, and data limitations with observational studies. Analyses were conducted between March 16, 2018 and May 25, 2021.

**Table 1:** Trial characteristics.

Study	Years of Study	Location	Population	Intervention	Control	Girls <18 years Available for Analysis	Women ≥18 years Available for Analysis
Adu-Afarwuah	2009–2011	Somanya-Kpong, Ghana	Pregnant women aged ≥18 years who were ≤20 weeks gestation; GA confirmed by ultrasound	MMN, LNS	IFA	0	1298
Ashorn	2011–2013	Mangochi district, Malawi	Pregnant women aged ≥15 years who were ≤20 weeks gestation; GA confirmed by ultrasound	MNN, LNS	IFA	131	1011
Belizan	1987–1989	Rosario, Argentina	Nulliparous women <20 weeks gestation with singleton pregnancies; GA confirmed by ultrasound	Calcium	Placebo	179	988
Bhutta	2002–2004	Bilal Colony, Karachi; Kot Diji district, rural Sindh	Pregnant women <16 weeks gestation; GA confirmed by ultrasound	MMN	IFA	64	2314
Christian (NNIPS-3)^1^	1998–2001	South eastern plains district, Sarlahi, Nepal	Pregnant women with newly identified pregnancy by urine test; GA calculated from LMP	MMN (including Vit A), FA-Fe-zinc-Vit A, IFA-Vit A, FA-Vit A	Vit A	643	3503
Dewey (Rang-Din Nutrition Study)^1^	2011–2012	Badarganj and Chirirbandar subdistricts, Bangladesh	Pregnant women ≤20 weeks gestation; GA calculated from LMP	LNS	IFA	643	3338
Fawzi	2001–2004	Dar es Salaam, Tanzania	Pregnant women estimated to be 12–27 weeks gestation; GA calculated from LMP	MMN	IFA	10	8068
Friis	1996–1997	Harare, Zimbabwe	Pregnant women 22–36 weeks gestation; GA calculated from LMP	MMN	IFA	78	698
Huybregts	2006–2008	Houndé health district, Burkina Faso	Pregnant women with formal pregnancy test completed; GA confirmed by ultrasound	MMN	LNS	138	1130
Kaestel	2001–2002	Bissau, Guinea-Bissau	Pregnant women <37 weeks gestation; GA calculated from LMP	MMN	IFA	134	1692
Osrin	2002–2004	Dhanusha and Mahottari districts, Nepal	Pregnant women 12–20 weeks gestation with a singleton pregnancy; GA confirmed by ultrasound	MMN	IFA	102	1098
Persson (MINIMat)	2001–2003	Matlab, Bangladesh	Pregnant women <14 weeks gestation who were confirmed by urine test; LMP used to calculate GA, though ultrasound was used to confirm GA <14 weeks at admission	MMN	IFA	263	4124
Roberfroid	2004–2006	Houndé health district, Burkina Faso	Pregnant women irrespective of gestational age; GA confirmed by ultrasound	MMN	IFA	155	1176
Shankar (SUMMIT)^1^	2001–2004	Lombok, Nusa Tenggara Barat Province, Indonesia	Pregnant women of any gestational age confirmed by physical exam or pregnancy test; GA calculated from LMP	MMN	IFA	1095	1605
West (JiVitA-3)^1^	2007–2012	Gaibandha and Rangpur districts, Bangladesh	Pregnant women with newly identified pregnancy by urine test; GA calculated from LMP	MMN	IFA	4731	24,397
West (JiVitA-1)^1^	2001–2007	Gaibandha and Rangpur districts, Bangladesh	Pregnant women aged 13–43 years, confirmed by urine test; GA calculated from LMP	Vit A, beta-carotene	Placebo	11,004	31,867
West (NNIPS-2)^1^	1994–1997	Sarlahi district, Nepal	Married women of childbearing age who became pregnant; GA calculated from LMP	Vit A, beta-carotene	Placebo	1072	14,316
WHO	2001–2003	Argentina, Egypt, India, South Africa, Peru, Vietnam	Nulliparous, normotensive pregnant women <20 weeks gestation with low calcium intake (<600 mg/day); GA determined by “best obstetric estimate” which included ultrasound if required by attending obstetrician	Calcium	Placebo	787	7468
Zagre^1^	2004–2006	Maradi, Niger	Pregnant women with pregnancy confirmed by pregnancy test after experiencing amenorrhoea for <12 weeks; GA calculation not reported	MMN	IFA	399	3258
Zeng^1^	2002–2006	Shaanxi Province, China	Pregnant women ≤28 weeks gestation; GA calculated from LMP	MMN	IFA	38	5734
TOTAL						21,666	119,083

1 Cluster-randomized trial (all other trials are individually randomized). Fe= iron; GA=gestational age; IFA= iron-folic acid; LMP= last menstrual period; LNS= lipid-nutrient supplement; MMN= multiple-micronutrient; Vit *A*= vitamin A.

### Outcomes and covariates

All outcomes, covariates of interest, and statistical methods were specified *a priori*. Maternal age groups were selected and categorised into biologically meaningful subgroups for adolescents (10–14 years, 15–17 years, 18–19 years) and women (20–29 years, 30–39 years, and 40+ years), as determined by global guidance and our study expert advisory group.^20^^,^^21^ Outcomes and covariates of interest were selected through consensus: based on expert opinion of the consortium co-investigators, advisory panel, and trial collaborators, with consideration given to feasibility, given the time and data management resources that a longer list would necessitate. Outcomes included birthweight (grams), LBW (<2500 gs), gestational age (weeks), preterm birth (<37 weeks), SGA (<10th centile, based on Intergrowth Standards), stillbirth, perinatal mortality, neonatal mortality, maternal hemoglobin (Hb), and maternal anemia (third trimester Hb <11.0 g/dL). Selected covariates measured at enrolment included gestational age at enrolment, maternal Hb at baseline, height and weight at baseline, parity, maternal education, and residential location of participant (urban or rural). We report individual trials’ method of assessment of gestational age in Table 1. Briefly, 7 out of 20 trials used ultrasonography, while the remainder calculated gestational age using the woman's first date of her last menstrual period. To categorize underweight/overweight and low stature among adolescents (up to age 19), the World Health Organization (WHO) age and sex-specific BMI-for-age and height-for-age growth charts were used as the reference. For women above age 19, we used the following cut-offs: (i) underweight: BMI <18.5, (ii) overweight: BMI ≥25.0, (iii) low stature: <152 cm.^22^ Selected covariates measured post-enrolment included number of antenatal care (ANC) visits and skilled birth attendance (SBA).

### Statistical analysis

For all analyses, participants from control and intervention arms were pooled (to ensure the largest possible sample size) and intervention status was included as a fixed effect. Sensitivity analyses conducted in duplicate by ECK and NA of control arm estimates only showed negligible differences (within 0.001) in parameter estimates when compared to the pooled control and intervention analyses. We thus opted for analyses based on the larger pooled sample size.

Summary statistics (frequencies/proportions, means/standard deviation (SD)) were calculated to examine baseline characteristics by maternal age groups. Adjusted means and prevalence of maternal and newborn outcomes were estimated with generalised linear models or log binomial regression models, respectively, with appropriate standard errors (SE) and 95% confidence intervals (CIs). Estimates were adjusted for fixed study effects, intervention given, maternal education and parity. Within-study clustering was accounted for by including trial as fixed effect in the models. All analyses were also performed stratified by region (Africa (*N* = 8 trials) and Asia (*N* = 10 trials)) when possible; 2 calcium supplementation trials were conducted outside of these geographies (in Argentina) and thus were not included in the analyses stratified by region. Some outcomes were not reported separately for Africa and Asia due to small sample sizes which led to model convergence issues. Minimum criteria for stratified analyses included: (i) for continuous outcomes, sample size ≥30 women, given the behavior and distribution of continuous outcomes which were approximately normal beyond that threshold, (ii) for birth outcomes (preterm, LBW, SGA) and maternal anemia, sample size ≥200, given that the prevalence of these conditions was high (10%−30% across trials) and (iii) for mortality outcomes (stillbirth, perinatal and neonatal mortality), sample size ≥500, given the rarity of these outcomes. Unstable estimates are denoted by red text within the tables. Applying these criteria, the 10–14 and 40+ groups were excluded entirely from analyses by region.

We fitted log binomial regression models using a log link function via a single-step model with age as a categorical variable and intervention type, maternal education, and parity as covariates to estimate adjusted relative risks (RR) of age effects on outcomes. Several other covariates were considered but not included due to extensive missing data (up to 73%) or a lack of notable variation across age groups (Appendix; Table S1). We conducted a complete case analysis without imputation. Model diagnostics were consulted as appropriate and parameters were estimated with SE and 95% CIs. For all analyses, HIV-positive women and multiple births were excluded. SAS version 9.4 and Stata version 15.1 were used to conduct analyses.

Ethical approval of the study was obtained through the Hospital for Sick Children's Research Ethics Board.

### Role of the funding source

The funders had no role in the procurement of data, access to data, or decision to submit for publication. NA, ECK, and SC had access to the study dataset and ZAB decided to submit the study for publication.

## Results

**Figure 1:**
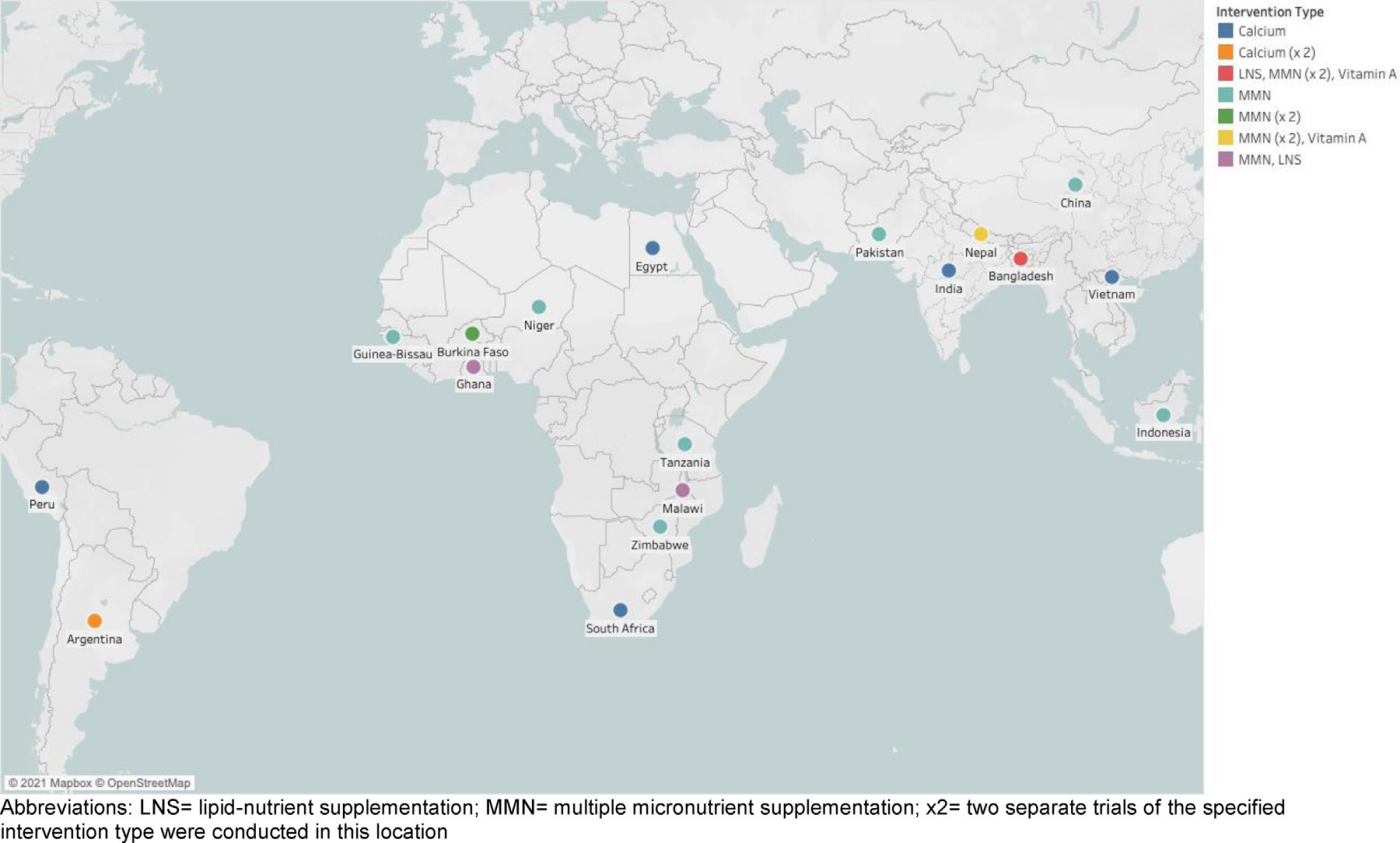
Map of included trials, disaggregated by intervention type.

The 20 included trials are detailed in Table 1 and shown in Figure 1. These trials represent 140,749 eligible participants, of whom 21,666 were girls less than 18 years of age and 119,083 were women 18 or above. There were 15 MMN supplementation trials (2 of which included a lipid-nutrient supplement (LNS) arm), 2 calcium supplementation trials, 2 vitamin A supplementation trials and 1 LNS trial. Maternal age distribution by trial and region can be found in the Appendix (Appendix; Tables S2–4). The age groups with the smallest proportions of mothers were those 40+ years and 10–14 years, which comprised 0.7% and 1.8% of the entire sample, respectively. The largest age group was 20–29 year olds, which made up 54.4% of the entire sample. It should also be noted that three trials contributed 62.1% of the sample (JiVitA-1, JiVitA-3, and NNIPS-2). Additionally, trials conducted in Africa contributed only 13.8% of all available data.

The distribution of mothers’ sociodemographic characteristics stratified by age group and region is shown in the Appendix (Table S5) for available variables. Older mothers had lower levels of education: 74.3% (95% CI; 72.6%−76.0%) of mothers aged 10–14 had some education compared to 25.2% (22.5%−28.0%) of those aged 40+. Nearly all mothers were married (99%), and most resided in rural areas (>90%). Parity increased with age: 3.6% (2.9−4.3%) of girls aged 10–14 were multiparous compared with 30.9% (30.3−31.6%) of girls aged 18–19 and 95.7% (94.4−97.0%) of women 40+ years. Access to clean water was high (>95%) and access to improved sanitation was around 50% across all age groups. When comparing regional differences, African girls (15–17 years) were less educated and had fewer children than Asians. Asian mothers generally had better access to improved sanitation.

Table S6 (Appendix) illustrates mothers’ delivery, nutrition, and healthcare characteristics**.** Gestational age at baseline appeared to increase slightly with age: girls 10–14 years had a mean (SD) gestational age of 11.8 (5.1) weeks and women 40+ years enrolled at a mean of 14.7 (7.2) weeks. Anemia at baseline was more prevalent among older women: around half of women aged 18–40+ had anemia compared to only a quarter of girls aged 10–14 years. Given that anthropometry was done at baseline (i.e. in the first trimester or generally early in pregnancy), height and weight can be assumed to reflect pre-pregnancy conditions. Prevalence (95% CI) of underweight increased with maternal age: from 0.6% (0.01−1.2%) of girls aged 10–14 years to 4.3% (3.9−4.6%) of girls 18–19, to around a quarter of women aged 20 and above. Overweight prevalence reached nearly 10% (7.5−12.0%) among the youngest girls and was highest (15%) for women aged 30+ years. Low stature afflicted 24.2% (21.0−27.4%) of girls aged 10–14 years and around 40% of mothers among all other age groups. Mean number of ANC visits generally increased and SBA coverage decreased with age. Mean gestational age at trial enrolment and anemia prevalence were higher for African mothers. More Asian mothers were underweight and had short stature, while more African mothers were overweight. African mothers generally attended more ANC visits and there was no difference in SBA by region.

**Figure 2:**
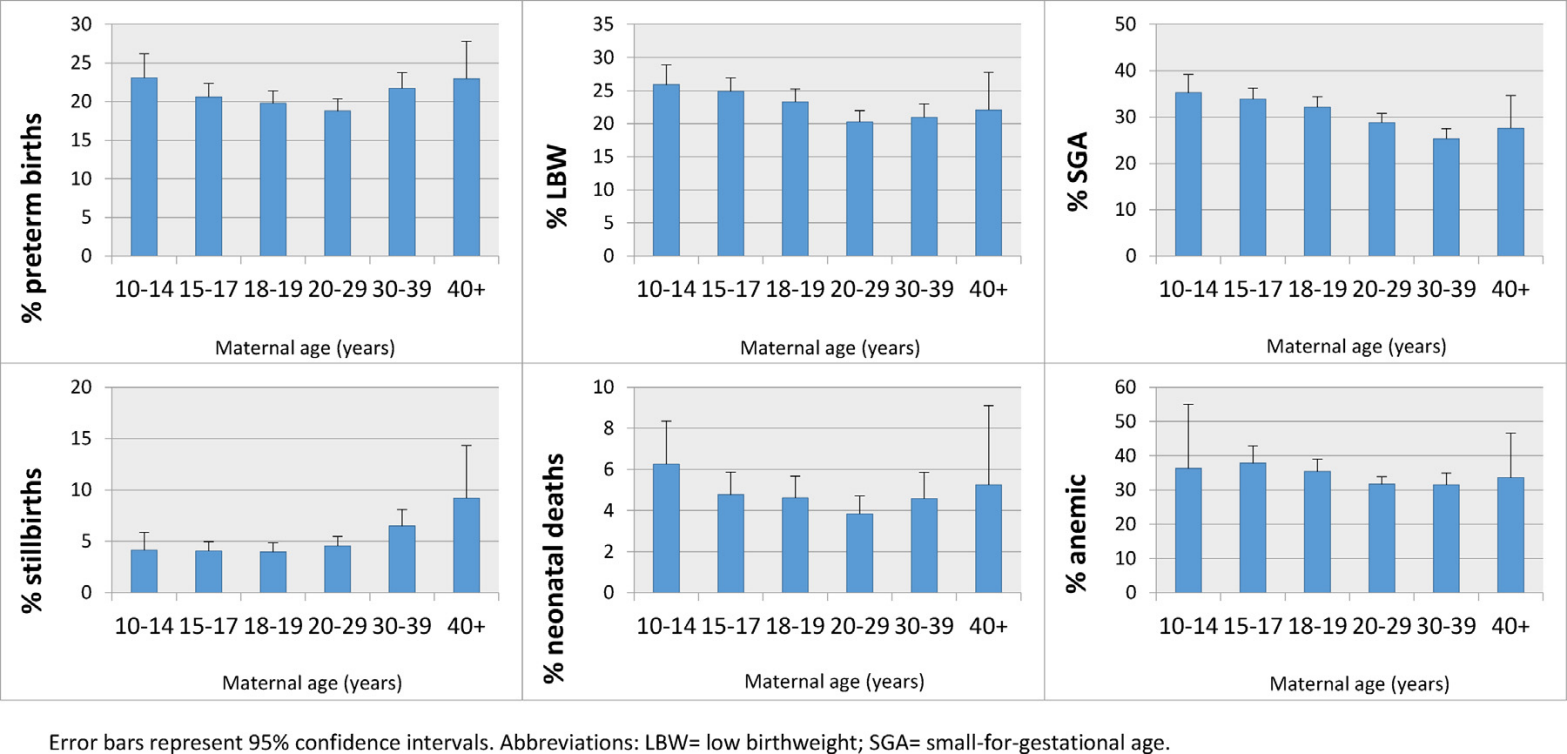
Prevalence of (a) preterm birth (%), (b) low birthweight (%), (c) small-for-gestational-age (%), (d) stillbirth (%), (e) neonatal mortality (%) and (f) maternal anemia (%), stratified by maternal age group.

Relationships between maternal age group and key outcomes are displayed in the Appendix (Table S7). Complete adjustment for trial design, intervention, and available confounders (education, parity) did not change the trends and we illustrate the adjusted estimates (adjusted means and proportions) in Figure 2. Prevalence of preterm births (Figure 2**a**) followed a U-shaped pattern. The outcome occurred most frequently in the youngest mothers (10–14 years) (23.1%; 95% CI: 21.6%−24.7%), and then declined until age 20–29, after which it increased again; mothers 40+ years had the second highest prevalence (22.9%; 20.7−25.5%). This pattern held for women residing in Asia, while preterm births appeared to decline until age 30–39 in Africa (Appendix; **Figure S1**). LBW (Figure 2**b**) followed the same U-shaped pattern: 25.9% (24.5−27.5%) of mothers aged 10–14 years had LBW babies compared to 20.3% (19.4−21.1%) of mothers aged 20–29 years and 22.0% (19.4−25.1%) of those 40+ years. Mothers in Asia across most age groups had a higher prevalence of LBW than mothers in Africa (Appendix; **Figure S2**). SGA prevalence was also highest in the youngest mothers and decreased until age 30–39 years, after which it increased again (Figure 2**c**). For girls aged 10–14 years, SGA prevalence reached 35.2% (33.3−37.3%), while for women 30–39 years it was 25.3% (24.2−26.4%) and for women 40+ it was 25.1% (21.3−29.3%). This trend persisted among Asian mothers, while African mothers showed little variation in SGA by age (Appendix; **Figure S3**). Stillbirth prevalence (Figure 2**d**) was lower among adolescent mothers and increased monotonically with age; prevalence was highest for mothers age 40+ years (9.2%; 7.0−12.1%). Asian mothers followed similar patterns by age (Appendix; **Figure S4**). Neonatal mortality prevalence also followed a strong U-shaped pattern with age (Figure 2**e**). Among 10–14 year old mothers, 6.3% (5.3−7.4%) of newborns died, compared to 3.8% (3.4−4.3%) among mothers aged 20–29 years, and 5.3% (3.7−7.5%) among mothers 40+ years. Newborn deaths were lowest among mothers aged 20–29 in Asia (Appendix; **Figure S5**). Perinatal mortality followed similar trends as newborn mortality, and results are shown in the Appendix (**Figs. S6 and S7**). Maternal anemia prevalence appeared to decrease with age, though wide CIs (due to small sample sizes) among 10–14 and 40+ year old mothers challenge any meaningful inferences for those age groups (Figure 2**f**). Anemia appeared to be frequent among teenaged mothers in Asia (Appendix; **Figure S8**).

Adjusted RR of key outcomes by maternal age group are presented in Table 2. Compared to mothers 20–29 years, mothers who were 15–17 years and 18–19 years had a 15% (RR (95% CIs); 1.08–1.22) and 6% (1.02–1.10) higher risk of maternal anemia, respectively. Mothers in all other age groups examined had a higher risk of preterm birth than those aged 20–29 years, with risks being greatest for the youngest mothers (23%; 1.15–1.31). Mothers aged 15–17 and 18–19 years had a 11% (0.81–0.97) and 12% (0.80–0.97) lower risk of stillbirth, respectively, than mothers aged 20–29, while mothers aged 30–39 had a 43% (1.32–1.56) increased risk and mothers 40+ years had a 103% higher risk of stillbirth (1.56–2.64). Compared to the 20–29 group, mothers aged 10–14, 15–17, 18–19, and 30–39 years had 60% (1.35–1.91), 18% (1.06–1.31), 15% (1.04–1.28), and 25% (1.12–1.41) higher risk of perinatal mortality. For neonatal mortality, risks were also greatest for 10–14 year old mothers, reaching 63% (1.40–1.90) increased risk. Higher risks were also observed for mothers aged 15–17, 18–19, and 30–39 years. Compared to the 20–29 group, risks of both LBW and SGA were highest among mothers aged 10–14, reaching 28% (1.22–1.35) and 22% (1.16–1.29) increased risk. Mothers aged 15–17 and 18–19 also had a greater risk of experiencing these adverse birth outcomes. For LBW, SGA, preterm birth, and neonatal mortality, a dose-response pattern emerged, whereby risks increased with declining maternal age. Table 1 also shows analyses with mothers 10–14 years as a reference group, and an assessment of <20 years vs >=20 year old mothers.

**Table 2:** Adjusted relative risks of key outcomes by maternal age group.

Maternalage group		Maternalanemia	Pretermbirth	Stillbirth	Perinatalmortality	Neonatalmortality	LBW	SGA
20–29		Ref.	Ref.	Ref.	Ref.	Ref.	Ref.	Ref.
10–14	RR (95% CI)	1.11 (0.83, 1.48)	1.23*** (1.15, 1.31)	0.92 (0.75, 1.12)	1.60*** (1.35, 1.91)	1.63*** (1.40, 1.90)	1.28*** (1.22, 1.35)	1.22*** (1.16, 1.29)
15–17	RR (95% CI)	1.15*** (1.08, 1.22)	1.10*** (1.06, 1.13)	**0.89** **(0.81, 0.97)**	**1.18** **(1.06, 1.31)**	1.25*** (1.13, 1.37)	1.23*** (1.19, 1.27)	1.18*** (1.14, 1.21)
18–19	RR (95% CI)	**1.06** **(1.02, 1.10)**	**1.05** **(1.02, 1.08)**	**0.88** **(0.80, 0.97)**	**1.15** **(1.04, 1.28)**	1.20*** (1.10, 1.31)	1.15*** (1.11, 1.18)	1.12*** (1.09, 1.15)
30–39	RR (95% CI)	0.99 (0.96, 1.03)	1.15*** (1.12, 1.19)	1.43*** (1.32, 1.56)	**1.25** **(1.12, 1.41)**	**1.19** **(1.08, 1.31)**	1.03 (0.99, 1.07)	0.88*** (0.85, 0.91)
40+	RR (95% CI)	1.03 (0.90, 1.17)	1.22*** (1.10, 1.35)	2.03*** (1.56, 2.64)	1.36 (0.90, 2.06)	1.37 (0.97, 1.94)	1.09 (0.96, 1.23)	0.96 (0.84, 1.08)
10–14		Ref.	Ref.	Ref.	Ref.	Ref.	Ref.	Ref.
15–17	RR (95% CI)	1.04 (0.77, 1.38)	**0.89** **(0.84, 0.95)**	0.97 (0.80, 1.18)	**0.74** **(0.62, 0.87)**	**0.77** **(0.66, 0.89)**	0.96 (0.91, 1.01)	0.96 (0.91, 1.01)
18–19	RR (95% CI)	0.96 (0.72, 1.28)	0.86*** (0.80, 0.91)	0.96 (0.79, 1.17)	**0.72** **(0.61, 0.85)**	0.74*** (0.63, 0.86)	0.90*** (0.85, 0.94)	**0.91** **(0.87, 0.96)**
20–29	RR (95% CI)	0.90 (0.68, 1.21)	0.82*** (0.77, 0.87)	1.09 (0.90, 1.33)	0.62*** (0.52, 0.74)	0.61*** (0.53, 0.72)	0.78*** (0.74, 0.82)	0.82*** (0.78, 0.86)
30–39	RR (95% CI)	0.90 (0.67, 1.20)	0.94 (0.88, 1.01)	1.56*** (1.27, 1.93)	**0.78** **(0.64, 0.96)**	**0.73** **(0.61, 0.87)**	0.81*** (0.76, 0.86)	0.72*** (0.68, 0.76)
40+	RR (95% CI)	0.93 (0.68, 1.27)	0.99 (0.88, 1.12)	2.21*** (1.60, 3.06)	0.85 (0.54, 1.33)	0.84 (0.58, 1.22)	**0.85** **(0.74, 0.97)**	**0.78** **(0.68, 0.90)**
<20		Ref.	Ref.	Ref.	Ref.	Ref.	Ref.	Ref.
≥20	RR (95% CI)	0.91 (0.82, 1.02)	1.00 (0.95, 1.05)	1.59*** (1.40, 1.81)	0.92 (0.78, 1.09)	0.87 (0.76, 1.01)	0.85*** (0.81, 0.90)	0.81*** (0.77, 0.85)

**Abbreviations:** CI = Confidence interval, LBW = Low birth weight, Ref = Reference group, RR = Relative Risk, SGA = Small-for-gestational age. Bold = *p* < 0.05 *** = *P* < 0.0001.

## Discussion

Findings from this study show that adolescent mothers, particularly the youngest mothers, are at a greater risk of experiencing adverse birth outcomes than older women. The exception to this was stillbirths, which were more common among older mothers (30+ years). Most outcomes examined followed a U-shaped pattern by age, whereby the youngest and oldest mothers experienced the worst outcomes, and mothers aged 20–29 years had the lowest risk. Within the cohort data, adverse outcomes were highly prevalent, reaching 35%, 26%, and 23% for SGA, LBW, and preterm births among the youngest adolescent mothers, respectively. Though our focus was on the adolescent group, the analysis also revealed that for the oldest mothers (40+ years) outcomes are unfavorable too, especially for SGA, preterm birth, LBW, stillbirths, perinatal and newborn mortality. Regional differences existed and most outcomes were worse in Asian versus African mothers. This could be due to the differing baseline characteristics between the populations, such as the higher rates of underweight and low stature among Asian women.^22^

There is some existing evidence to support our findings. The previously referenced meta-analysis found that nulliparous women <18 years had the highest odds of preterm birth, SGA, neonatal mortality, and infant mortality.^15^ The authors conducted a sensitivity analysis that pointed to even higher odds for mothers <16 years, particularly for preterm births, though the results were not conclusive (which was likely due to small sample size). A more recent meta-analysis of 18 studies examining complications associated with adolescent childbearing in Sub-Saharan Africa found that adolescent mothers (<17 years) had an increased risk of adverse neonatal and maternal outcomes including preeclampsia/eclampsia, LBW, preterm birth, perinatal death and maternal death.^23^ Other studies have also demonstrated higher rates of preterm birth and mortality outcomes for young mothers.^17^^,^^24^, ^25^, ^26^, ^27^ A larger study from the Brazilian Network for Surveillance of Severe Maternal Morbidity also found that maternal near miss or maternal death was significantly higher among older women (>35 years).^28^

Results from both our crude and adjusted analyses (for parity and education) showed similar patterns, though covariate adjustment did attenuate estimates slightly. Our study therefore supports the narrative that worse birth outcomes experienced by very young and older mothers are likely related to both biology and environmental/socioeconomic conditions experienced by these populations.^25^^,^^27^^,^^29^, ^30^, ^31^ However, due to the limited set of contextual covariates available in our datasets, we could not thoroughly explore or confirm these trends. Yet the finding is consistent with other cross-sectional reviews that found adverse pregnancy and birth outcomes among young mothers, even after adjusting for social and economic confounders.^24^^,^^25^^,^^40^^,^^32^, ^33^, ^34^, ^35^, ^36^, ^37^, ^38^, ^39^ A meta-analysis of 118 DHS datasets from 55 LMICs found that adolescent and young mothers experienced poorer newborn health and mortality outcomes; these findings persisted even after controlling for maternal, paternal, household, and social factors.^15^ Similar to our study, authors also found improvements in child health outcomes as the age of the mother increased to 27–29 years. Authors concluded that both social mechanisms and biological maturity play a role in birth outcomes. These conclusions are further supported by the lack of adverse outcomes found when considering young fathers. If being a young mother is simply a marker for poor social conditions, we might expect to find similar adverse child outcomes among children of young fathers. However, two studies that sought to examine differential effects of maternal and paternal conditions on child health, as a method of distinguishing between biological and social mechanisms, did not corroborate this^15^^,^^41^; suggesting that factors beyond socioeconomic determinants could play a key role. We posit that in addition to nutrition partitioning, biological immaturity of young mothers, for example resulting in insufficiency in maturity of the uterine and cervical blood supply, may also be linked to adverse birth outcomes seen in this population.

Indeed, a plausible and well-referenced biological mechanism of action is incomplete physical and sexual maturation, coupled with higher nutritional demands during adolescence. In this situation, a young adolescent girl is at a much greater disadvantage than an older woman with adequate nutrient status who may not experience competing demands between mother and fetus.^6^^,^^42^ Height and pelvic growth are not complete until close to two years following first menstruation, underscoring one of several vulnerable periods in an adolescent girl's reproductive years.^24^ Maternal stunting and small pelvic size have been associated with poor fetal growth and adverse obstetric outcomes, including obstructed labor and asphyxia of the infant.^22^ Additional evidence has indicated that a mother's undernutrition can lead to smaller placental mass, poor vascularization, and less nutrient transfer to the fetus,^43^^,^^44^ and some adolescent mothers weigh significantly less, with lower BMI, than adult mothers.^45^ Anemia in pregnancy has been associated with increased risks of LBW, preterm birth, perinatal mortality, and neonatal mortality,^46^ and examinations of adolescent-specific populations have confirmed these findings.^47^^,^^48^ Much less is known about multiple micronutrient deficiencies in this population. Taken together, younger mothers, particularly those who have micronutrient deficiencies and other types of malnutrition prevalent in LMICs, are more likely to experience adverse outcomes relating directly to parturition and poor growth and development of the fetus.

While biology is likely to play a role, there may be socioeconomic and other social determinants that contribute to the observed risks for adolescents. Lack of empowerment of adolescent mothers, often due to early marriage, incomplete education, poor access to financial, healthcare and other resources, low decision-making ability, and other context-specific social and gender norms, contribute to reduced agency.^49^ Each of these domains of empowerment – alone or together – could lead to adverse pregnancy outcomes through a lack of knowledge of best practices (e.g. relating to nutrition), inadequate resources to exercise these practices, or poor access to quality maternal and other health services because of financial, social, and other barriers. There is evidence to support the link between adolescent wellbeing, pregnancy, and undesirable pregnancy and birth outcomes.^12^^,^^45^ A meta-analysis looking at factors influencing the utilization of health services by adolescent mothers in LMICs^50^ found that both maternal and paternal education were among the most important factors, along with wealth, media exposure, and urban/rural residence. Though good quality ANC may not protect adolescents from the biological effects discussed above, authors also noted positive associations between ANC and SBA, and ANC/SBA and postnatal care, underscoring the benefits of prenatal care for adolescent girls.^50^ Though our findings clearly demonstrate differences in pregnancy and neonatal outcomes by age, we unfortunately could not discern the relative impact of biological versus environmental factors with the available data.

The association between advanced maternal age and increased risk of adverse birth and maternal outcomes has been found in many studies from high-income countries.^51^, ^52^, ^53^, ^54^, ^55^ Our study provides new data from LMICs to support these findings, particularly for SGA, LBW, preterm births, stillbirths, perinatal mortality and newborn mortality outcomes. These data require immediate attention and action from global agenda setters and country goverments; specifically, tailored interventions to support healthy antenatal care and delivery practices among older mothers are essential. Distinguishing the relative contributions of social conditions and biology in older mothers will require additional research. It is worth noting that many of these trials are now 15+ years old, underscoring a sociodemographic profile of families and communities that has likely changed over time and may have differentially influenced outcomes, particularly for older mothers. Future research that examines this cohort effect would shed light on how the impacts of environmental factors may vary over time.

The analysis had several limitations. As discussed above, we were unable to disentangle the relative contribution of biology versus socioeconomic conditions to the relationship between young age and adverse pregnancy and birth outcomes since we lacked a full set of individual and household-level confounders to adjust for. Thus, the observed associations may be subject to residual confounding (e.g. due to mental health status, empowerment and agency, or others). There is a possibility of some misclassification between stillbirths and early neonatal mortality,^56^ though this risk is likely to be lower than usual given that the women were being followed in trials and surveillance systems were probably more stringent. The majority of trials included (13/20) assessed gestational age using maternal recall to determine a woman's last menstrual period, which is an important source of error and could result in either over- or under-estimating preterm birth prevalence. However, a sensitivity analysis whereby only trials with ultrasound-confirmed gestational age were included revealed the same U-shaped pattern for preterm births. We did not consider imputing covariate information since, often, missingness was from whole studies or there was >30% missing data and thus imputation would be inappropriate. Similarly, because trials from Africa comprised such a small proportion of our total sample, we may have been unable to discern true differences in outcomes and baseline characteristics by region, especially for the youngest and oldest mothers, which were far fewer in number when compared to the middle range of mothers. Trials that were not conducted in Asia or Africa (calcium supplementation trials) were dropped from the regional analysis entirely, underscoring a gap in evidence for South America and other geographies that were not represented by our data (e.g. the Middle East). Because the supplementation trials included in this analysis were not targeting adolescents exclusively, and a number of the outcomes examined are rare at a population level, this made statistical power problematic. In a best-case scenario, a cohort analysis such as this one could be repeated with large trials that span various geographies and that consist only of adolescent mothers. Also, given the pooled trials had different interventions, varying trial designs and diverse population characteristics, heterogeneity does exist. However, given the scope and objectives of this analysis, we feel its impact on overall inferences is limited.

Despite limitations, this analysis suggests that there are substantial differences in adverse birth and neonatal outcomes by maternal age. Though results are mostly generalizable to LMIC settings, more research is needed to determine if the same patterns exist in high-income countries. Strengths include the use of good quality IPD from prospective RCTs. To estimate outcomes, we controlled for education and parity, two of the most relevant covariates when considering adolescent pregnancy.^15^ Additionally, we have disaggregated maternal age by 6 subgroups compared to the typical cut-off of <18 and ≥18 years, and found that a trend exists for adolescents that puts the youngest mothers at greatest risk. We found that risks also exist for women who are nearing the end of their reproductive years. These findings have important implications for in-country policy and programming initiatives. Adolescent mothers are at a clear disadvantage, both from a biological predisposition for high-risk pregnancies and because of status in a socioeconomic and cultural sense. Targeted strategies should be used to mitigate these risks, especially where contraceptive use is low and adolescent pregnancies are high. Indirect approaches to delay pregnancy may include the initiation of female empowerment programmes, community sensitization to adolescent sexual and reproductive health and rights, and the inclusion of adolescent boys in educational initiatives. Direct approaches may work to increase the provision of adolescent-friendly health services, including the promotion of contraceptive awareness and uptake through schools and other delivery platforms that can reach out-of-school adolescents. There is also a need to reach adolescents with quality ANC. A group prenatal care model is one such platform that may benefit and support vulnerable groups of adolescent mothers and improve health outcomes, education, and adherence to pregnancy recommendations.^57^ In addition, prenatal care that includes men has been shown to be effective at improving pregnancy outcomes.^58^ On the contrary, efforts to understand advanced age pregnant women, their risk factors, and interventions to prevent adverse outcomes, are also paramount and evidence from this study could be used to better study this neglected group in LMICs.

In conclusion, our analysis demonstrates measurable differences between adolescents and older mothers in LMICs for key birth and mortality outcomes. Though we have yet to uncover the reasons behind the differences, whether biological or environmental, these findings warrant further investigation based on the high prevalence of adolescent pregnancy in LMICs. In the meantime, specific interventions for adolescent and older mothers (40+ years) will be necessary to achieve fewer child deaths and better pregnancy outcomes on a global level. We call on governments, donors and policy-makers to focus on these vulnerable female populations (younger and older prospective mothers) in LMICs to ensure that health pre-pregnancy, delivery and postnatal care is accessible for women and their children.

## Contributors

ZAB conceptualised the study and wrote the proposal for funding with NA. ECK managed all coordination activities of the study, including with the wider Consortium members. PT performed the systematic review and managed, cleaned, and merged the received individual participant data, with oversight from NA and ECK. SC cleaned/managed all the individual-level data and provided substantial oversight on all analyses, which were conducted by NA and ECK. Authors named as members of the Global Young Women's Nutrition Investigators Group made substantial intellectual contributions throughout the study as Technical Advisors or Steering Committee members, and/or provided individual-level data. NA and ECK drafted and revised the manuscript with input from all co-authors. All authors can take responsibility for the integrity of the data and accuracy of the data analysis. NA, ECK, and SC had access to the study dataset and ZAB decided to submit the study for publication.

### Funding

10.13039/100000865Bill and Melinda Gates Foundation(Grant No: OP1137750).

### Data sharing

Data sharing agreements between The Hospital for Sick Children and relevant institutions were put into place in order to transfer individual participant data for each included trial.

## Declaration of interests

We declare no competing interests.

## Appendix Supplementary materials

Image, application 1
