# Supplementary material for: Characteristics and birth outcomes of pregnant adolescents compared to older women: An analysis of individual level data from 140,000 mothers from 20 RCTs
Source: eClinicalMedicine. 2022 Feb 26;45:101309. doi: 10.1016/j.eclinm.2022.101309 (PMC8885463; doi:10.1016/j.eclinm.2022.101309)
Supplement: Supplementary file 1 [file mmc1.docx]

**Characteristics and birth outcomes of pregnant adolescents compared to older women: an analysis of individual level data from 140,000 mothers from 20 RCTs**

Supplementary Material

**Appendix 1: Additional Study Data and Results**

**Table S1:** Distribution of potential covariates by age group and % missing.

|  |  | 10-14 years | 15-17 years | 18-19 years | 20-29 years | 30-39 years | 40+  years |
| --- | --- | --- | --- | --- | --- | --- | --- |
| % urban | % | 0.28 | 1.61 | 8.67 | 11.46 | 11.18 | 9.74 |
|  | Total N | 2565 | 19,101 | 22,874 | 76,537 | 18,677 | 995 |
|  | N missing | 64 | 723 | 1336 | 6443 | 947 | 40 |
|  | % missing | 2.50 | 3.79 | 5.84 | 8.42 | 5.07 | 4.02 |
| % married | % | 100.00 | 99.43 | 99.11 | 99.22 | 99.47 | 98.92 |
|  | Total N | 2565 | 19,101 | 22,874 | 76,537 | 18,677 | 995 |
|  | N missing | 196 | 3614 | 6318 | 24,472 | 5516 | 349 |
|  | % missing | 7.64 | 18.9 | 27.6 | 32.0 | 29.5 | 35.1 |
| % access to clean water | % | 99.92 | 99.87 | 99.64 | 99.15 | 98.62 | 98.25 |
|  | Total N | 2565 | 19,101 | 22,874 | 76,537 | 18,677 | 995 |
|  | N missing | 177 | 3426 | 7215 | 30,211 | 7443 | 424 |
|  | % missing | 6.90 | 17.9 | 31.5 | 39.5 | 39.9 | 42.6 |
| % access to improved sanitation | % | 47.68 | 57.05 | 63.32 | 57.09 | 52.40 | 43.99 |
|  | Total N | 2565 | 19,101 | 22,874 | 76,537 | 18,677 | 995 |
|  | N missing | 174 | 3305 | 6949 | 28,917 | 7034 | 404 |
|  | % missing | 6.78 | 17.3 | 30.4 | 37.8 | 37.7 | 40.6 |
| Maternal height | Mean | 149.59 | 150.85 | 152.00 | 153.58 | 153.93 | 153.97 |
|  | Total N | 2565 | 19,101 | 22,874 | 76,537 | 18,677 | 995 |
|  | N missing | 1876 | 9967 | 9204 | 28,998 | 7052 | 417 |
|  | % missing | 73.1 | 52.2 | 40.2 | 37.9 | 37.8 | 41.9 |
| Maternal weight | Mean | 43.67 | 45.50 | 47.43 | 49.43 | 51.34 | 51.62 |
|  | Total N | 2565 | 19,101 | 22,874 | 76,537 | 18,677 | 995 |
|  | N missing | 1882 | 9994 | 9241 | 29,382 | 7153 | 425 |
|  | % missing | 73.4 | 52.3 | 40.4 | 38.4 | 38.3 | 42.7 |
| Maternal Hb | Mean (SD) | 11.81 | 11.16 | 10.89 | 10.90 | 10.83 | 10.89 |
|  | Total N | 2565 | 19,101 | 22,874 | 76,537 | 18,677 | 995 |
|  | N missing | 2414 | 16,672 | 18,257 | 61,699 | 14,676 | 757 |
|  | % missing | 94.1 | 87.3 | 79.8 | 80.6 | 78.6 | 76.1 |
| Maternal anemia (Hb <11.0 g/L) | % | 23.84 | 41.79 | 49.23 | 48.58 | 49.44 | 48.74 |
|  | Total N | 2565 | 19,101 | 22,874 | 76,537 | 18,677 | 995 |
|  | N missing | 2414 | 16,672 | 18,257 | 61,699 | 14,676 | 757 |
|  | % missing | 94.1 | 87.3 | 79.8 | 80.6 | 78.6 | 76.1 |
| Gestational age at enrolment | Mean (SD) | 11.81 | 12.88 | 14.00 | 13.64 | 13.84 | 14.65 |
|  | Total N | 2565 | 19,101 | 22,874 | 75,537 | 18,677 | 995 |
|  | N missing | 19 | 202 | 352 | 1283 | 295 | 32 |
|  | % missing | 0.74 | 1.1 | 1.5 | 1.7 | 1.6 | 3.2 |
| ANC visits | Mean (SD) | 0.48 | 0.84 | 1.09 | 1.21 | 1.21 | 1.04 |
|  | Total N | 2565 | 19,101 | 22,874 | 76,537 | 18,677 | 995 |
|  | N missing | 298 | 5030 | 9347 | 32451 | 8238 | 473 |
|  | % missing | 11.6 | 26.3 | 40.9 | 42.4 | 44.1 | 47.5 |
| % with SBA | % | 98.56 | 95.40 | 94.17 | 95.46 | 92.94 | 93.01 |
|  | Total N | 2565 | 19,101 | 22,874 | 76,537 | 18,677 | 995 |
|  | N missing | 554 | 4197 | 4994 | 18,791 | 4461 | 265 |
|  | % missing | 21.6 | 22.0 | 21.8 | 24.6 | 23.9 | 26.6 |

**Table S2:** Distribution of all women available for analysis by age group.

|  | Trial | Age Group | | | | | | |
| --- | --- | --- | --- | --- | --- | --- | --- | --- |
|  |  | **10-14** | **15-17** | **18-19** | **20-29** | **30-39** | **40+** | **Total** |
| 1 | Adu-Afarwuah | 0 | 0 | 105 | 822 | 346 | 25 | 1298 |
| 2 | Ashorn | 3 | 128 | 167 | 629 | 202 | 13 | 1142 |
| 3 | Belizan | 20 | 159 | 134 | 682 | 167 | 5 | 1167 |
| 4 | Bhutta | 4 | 60 | 145 | 1579 | 555 | 35 | 2378 |
| 5 | Christian (NNIPS-3) | 51 | 592 | 640 | 2258 | 566 | 39 | 4146 |
| 6 | Dewey (Rang-Din Nutrition Study) | 36 | 607 | 898 | 2084 | 339 | 17 | 3981 |
| 7 | Fawzi | 0 | 10 | 1287 | 5402 | 1323 | 56 | 8078 |
| 8 | Friis | 3 | 75 | 125 | 460 | 108 | 5 | 776 |
| 9 | Huybregts | 3 | 135 | 186 | 674 | 243 | 27 | 1268 |
| 10 | Kaestel | 3 | 131 | 255 | 1114 | 304 | 19 | 1826 |
| 11 | Osrin | 0 | 102 | 259 | 785 | 54 | 0 | 1200 |
| 12 | Persson (MINIMat) | 14 | 249 | 446 | 2501 | 1120 | 57 | 4387 |
| 13 | Roberfroid | 1 | 154 | 197 | 693 | 263 | 23 | 1331 |
| 14 | Shankar (SUMMIT) | 17 | 1078 | 1605 | 0 | 0 | 0 | 2700 |
| 15 | West (JiVitA-3) | 397 | 4334 | 5183 | 15502 | 3543 | 169 | 29128 |
| 16 | West (JiVitA-1) | 1927 | 9077 | 6845 | 20096 | 4665 | 261 | 42871 |
| 17 | West (NNIPS-2) | 13 | 1059 | 2372 | 9333 | 2449 | 162 | 15388 |
| 18 | WHO | 64 | 723 | 1231 | 5621 | 601 | 15 | 8255 |
| 19 | Zagre | 9 | 390 | 372 | 1959 | 869 | 58 | 3657 |
| 20 | Zeng | 0 | 38 | 422 | 4343 | 960 | 9 | 5772 |
|  | TOTAL | 2565 | 19101 | 22874 | 76537 | 18677 | 995 | 140749 |

**Table S3**: Distribution of women (Africa trials) available for analysis by age group.

|  | Trial | Age Group | | | | | | |
| --- | --- | --- | --- | --- | --- | --- | --- | --- |
|  |  | **10-14** | **15-17** | **18-19** | **20-29** | **30-39** | **40+** | **Total** |
| 1 | Adu-Afarwuah | 0 | 0 | 105 | 822 | 346 | 25 | 1298 |
| 2 | Ashorn | 3 | 128 | 167 | 629 | 202 | 13 | 1142 |
| 3 | Fawzi | 0 | 10 | 1287 | 5402 | 1323 | 56 | 8078 |
| 4 | Friis | 3 | 75 | 125 | 460 | 108 | 5 | 776 |
| 5 | Huybregts | 3 | 135 | 186 | 674 | 243 | 27 | 1268 |
| 6 | Kaestel | 3 | 131 | 255 | 1114 | 304 | 19 | 1826 |
| 7 | Roberfroid | 1 | 154 | 197 | 693 | 263 | 23 | 1331 |
| 8 | Zagre | 9 | 390 | 372 | 1959 | 869 | 58 | 3657 |
|  | TOTAL | 22 | 1023 | 2694 | 11753 | 3658 | 226 | 19376 |

**Table S4**: Distribution of women (Asia trials) available for analysis by age group.

|  | Trial | Age Group | | | | | | |
| --- | --- | --- | --- | --- | --- | --- | --- | --- |
|  |  | **10-14** | **15-17** | **18-19** | **20-29** | **30-39** | **40+** | **Total** |
| 1 | Bhutta | 4 | 60 | 145 | 1579 | 555 | 35 | 2378 |
| 2 | Christian | 51 | 592 | 640 | 2258 | 566 | 39 | 4146 |
| 3 | Dewey | 36 | 607 | 898 | 2084 | 339 | 17 | 3981 |
| 4 | Osrin | 0 | 102 | 259 | 785 | 54 | 0 | 1200 |
| 5 | Persson (MINIMat) | 14 | 249 | 446 | 2501 | 1120 | 57 | 4387 |
| 6 | Shankar (SUMMIT) | 17 | 1078 | 1605 | 0 | 0 | 0 | 2700 |
| 7 | West (JiVitA-3) | 397 | 4334 | 5183 | 15502 | 3543 | 169 | 29128 |
| 8 | West (JiVitA-1) | 1927 | 9077 | 6845 | 20096 | 4665 | 261 | 42871 |
| 9 | West (NNIPS-2) | 13 | 1059 | 2372 | 9333 | 2449 | 162 | 15388 |
| 10 | Zeng | 0 | 38 | 422 | 4343 | 960 | 9 | 5772 |
|  | TOTAL | 2459 | 17196 | 18815 | 58481 | 14251 | 749 | 111951 |

**Table S5:** Crude participant socio-demographic characteristics by maternal age group.

|  |  |  | 10-14 years | 15-17 years | 18-19 years | 20-29 years | 30-39 years | 40+  years |
| --- | --- | --- | --- | --- | --- | --- | --- | --- |
| % educated | Overall | % | 74.3 | 71.7 | 70.8 | 58.0 | 40.8 | 25.2 |
|  |  | 95% CI | 72.6-76.0 | 71.0-72.3 | 70.2-71.4 | 57.7-58.4 | 40.0-41.5 | 22.5-28.0 |
|  |  | Total N | 2532 | 18,718 | 22,288 | 73,682 | 17,602 | 931 |
|  | Asia | % | 74.1 | 73.0 | 69.9 | 52.7 | 34.5 | 19.5 |
|  |  | 95% CI | 72.4-75.8 | 72.3-73.7 | 69.3-70.6 | 52.3-53.1 | 33.7-35.4 | 16.6-22.4 |
|  |  | Total N | 2451 | 17,092 | 18,567 | 57,032 | 13,525 | 703 |
|  | Africa | % | 17.7 | 28.9 | 66.8 | 66.0 | 55.3 | 39.4 |
|  |  | 95% CI | 0.0-35.8 | 25.9-31.9 | 64.9-68.6 | 65.1-66.8 | 53.6-56.9 | 32.9-46.0 |
|  |  | Total N | 17 | 903 | 2490 | 11,031 | 3476 | 213 |
| % married | Overall | % | 100 | 99.4 | 99.1 | 99.2 | 99.5 | 98.9 |
|  |  | 95% CI | 100-100 | 99.3-99.5 | 99.0-99.3 | 99.1-99.3 | 99.3-99.6 | 98.1-99.7 |
|  |  | Total N | 2369 | 15,487 | 16,556 | 52,065 | 13,161 | 646 |
|  | Asia | % | 100.0 | 100.0 | 100.0 | 100.0 | 100.0 | 100.0 |
|  |  | 95% CI | 100-100 | 100-100 | 100-100 | 100-100 | 100-100 | 100-100 |
|  |  | Total N | 2355 | 14,878 | 14,760 | 43,227 | 10,342 | 496 |
|  | Africa | % | 100.0 | 85.4 | 91.8 | 95.4 | 97.5 | 95.3 |
|  |  | 95% CI | 100-100 | 82.6-88.2 | 90.5-93.0 | 94.9-95.8 | 96.9-98.1 | 92.0-98.7 |
|  |  | Total N | 14 | 609 | 1796 | 8838 | 2819 | 150 |
| % urban | Overall | % | 0.3 | 1.6 | 8.7 | 11.5 | 11.2 | 9.7 |
|  |  | 95% CI | 0.1-0.5 | 1.4-1.8 | 8.3-9.1 | 11.2-11.7 | 10.7-11.7 | 7.9-11.6 |
|  |  | Total N | 2501 | 18,378 | 21,538 | 70,094 | 17,730 | 955 |
|  | Asia | % | 0.0 | 0.5 | 1.1 | 1.8 | 1.7 | 1.7 |
|  |  | 95% CI | 0.0-0.1 | 0.4-0.6 | 0.9-1.2 | 1.7-1.9 | 1.5-1.9 | 0.8-2.7 |
|  |  | Total N | 2459 | 17,196 | 18,815 | 58,481 | 14,251 | 749 |
|  | Africa | % | 27.3 | 21.1 | 64.4 | 63.8 | 52.4 | 39.8 |
|  |  | 95% CI | 8.7-45.9 | 18.6-23.6 | 62.5-66.2 | 62.9-64.7 | 50.7-54.1 | 33.0-46.6 |
|  |  | Total N | 22 | 1023 | 2589 | 10,931 | 3312 | 201 |
| % multiparous | Overall | % | 3.6 | 13.8 | 30.9 | 74.4 | 93.8 | 95.7 |
|  |  | 95% CI | 2.9-4.3 | 13.3-14.3 | 30.3-31.6 | 74.1-74.7 | 93.4-94.2 | 94.4-97.0 |
|  |  | Total N | 2487 | 18,011 | 21,032 | 68,400 | 17,380 | 934 |
|  | Asia | % | 3.6 | 14.1 | 32.3 | 76.1 | 94.6 | 95.7 |
|  |  | 95% CI | 2.9-4.3 | 13.6-14.6 | 31.6-32.9 | 75.8-76.5 | 94.3-95.0 | 94.2-97.1 |
|  |  | Total N | 2454 | 17,167 | 18,758 | 58,190 | 14,177 | 740 |
|  | Africa | % | 7.7 | 9.6 | 21.3 | 69.0 | 95.1 | 98.4 |
|  |  | 95% CI | 0.0-22.2 | 7.4-11.8 | 19.5-23.0 | 68.1-69.9 | 94.4-95.9 | 96.6-100.0 |
|  |  | Total N | 13 | 685 | 2140 | 9528 | 3036 | 189 |
| % access to clean water | Overall | % | 99.9 | 99.9 | 99.6 | 99.2 | 98.6 | 98.3 |
|  |  | 95% CI | 99.8-100.0 | 99.8-99.9 | 99.5-99.7 | 99.1-99.2 | 98.4-98.8 | 97.2-99.3 |
|  |  | Total N | 2388 | 15,675 | 15,659 | 46,326 | 11,234 | 571 |
|  | Asia | % | 100.0 | 99.9 | 99.9 | 99.6 | 99.4 | 99.2 |
|  |  | 95% CI | 99.9-100.0 | 99.9-100.0 | 99.9-99.9 | 99.5-99.6 | 99.2-99.5 | 98.3-99.9 |
|  |  | Total N | 2376 | 15,164 | 15,023 | 42,952 | 9828 | 476 |
|  | Africa | % | 91.7 | 98.4 | 94.5 | 93.6 | 93.5 | 93.7 |
|  |  | 95% CI | 76.0-100.0 | 97.4-99.5 | 92.7-96.3 | 92.7-94.4 | 92.2-94.8 | 88.8-98.6 |
|  |  | Total N | 12 | 511 | 636 | 3374 | 1406 | 95 |
| % access to improved sanitation | Overall | % | 47.7 | 57.1 | 63.3 | 57.1 | 52.4 | 44.0 |
|  |  | 95% CI | 45.7-49.7 | 56.3-57.8 | 62.6-64.1 | 56.6-57.7 | 51.5-53.3 | 40.0-48.0 |
|  |  | Total N | 2391 | 15,796 | 15,925 | 47,620 | 11,643 | 591 |
|  | Asia | % | 47.9 | 58.5 | 65.0 | 59.5 | 56.2 | 47.2 |
|  |  | 95% CI | 45.8-49.9 | 57.7-59.3 | 64.3-65.8 | 59.0-60.0 | 55.2-57.2 | 42.8-51.7 |
|  |  | Total N | 2376 | 15,181 | 15,073 | 43,327 | 9993 | 487 |
|  | Africa | % | 20.0 | 21.5 | 33.0 | 32.7 | 29.5 | 28.9 |
|  |  | 95% CI | 0.0-40.2 | 18.2-24.7 | 29.8-36.1 | 31.3-34.1 | 27.3-31.7 | 20.1-37.6 |
|  |  | Total N | 15 | 615 | 852 | 4293 | 1650 | 104 |

Total N = effective sample size; Unstable estimates in red.

**Table S6:** Crude participant nutrition and healthcare characteristics by maternal age group at study enrolment.

|  |  |  | 10-14 years | 15-17 years | 18-19 years | 20-29 years | 30-39 years | 40+  years |
| --- | --- | --- | --- | --- | --- | --- | --- | --- |
| Mean gestational age (weeks) | Overall | Mean | 11.8 | 12.9 | 14.0 | 13.6 | 13.8 | 14.7 |
|  |  | SD | 5.1 | 6.1 | 6.7 | 6.3 | 6.7 | 7.4 |
|  |  | 95% CI | 11.6-12.0 | 12.8-13.0 | 13.9-14.1 | 13.6-13.7 | 13.8-13.9 | 14.2-15.1 |
|  |  | Total N | 2546 | 18,899 | 22,522 | 75,254 | 18,382 | 963 |
|  | Asia | Mean | 11.6 | 12.6 | 13.2 | 12.3 | 12.7 | 13.8 |
|  |  | SD | 5.0 | 6.0 | 6.5 | 5.9 | 6.3 | 7.5 |
|  |  | 95% CI | 11.4-11.8 | 12.5-12.7 | 13.1-13.3 | 12.3-12.4 | 12.6-12.8 | 13.2-14.3 |
|  |  | Total N | 2440 | 16,999 | 18,477 | 57,253 | 13,974 | 723 |
|  | Africa | Mean | 15.9 | 15.9 | 19.0 | 18.8 | 17.8 | 17.3 |
|  |  | SD | 6.8 | 7.5 | 6.4 | 6.4 | 6.7 | 6.8 |
|  |  | 95% CI | 12.8-18.9 | 15.5-16.4 | 18.7-19.2 | 18.7-18.9 | 17.5-18.0 | 16.4-18.2 |
|  |  | Total N | 22 | 1018 | 2680 | 11,698 | 3640 | 220 |
| Mean Hb (g/dL) | Overall | Mean | 11.8 | 11.2 | 10.9 | 10.9 | 10.8 | 10.9 |
|  |  | SD | 1.4 | 1.5 | 1.6 | 1.6 | 1.6 | 1.6 |
|  |  | 95% CI | 11.6-12.0 | 11.1-11.2 | 10.9-10.9 | 10.9-10.9 | 10.8-10.9 | 10.7-11.1 |
|  |  | Total N | 151 | 2429 | 4617 | 14,838 | 4001 | 238 |
|  | Asia | Mean | 12.0 | 11.3 | 11.3 | 11.3 | 10.9 | 10.8 |
|  |  | SD | 1.3 | 1.5 | 1.5 | 1.6 | 1.7 | 1.5 |
|  |  | 95% CI | 11.8-12.3 | 11.2-11.4 | 11.2-11.3 | 11.2-11.3 | 10.8-11.0 | 10.4-11.1 |
|  |  | Total N | 127 | 1707 | 2439 | 5478 | 1323 | 76 |
|  | Africa | Mean | 9.7 | 10.8 | 10.5 | 10.6 | 10.8 | 11.0 |
|  |  | SD | 1.9 | 1.6 | 1.6 | 1.6 | 1.6 | 1.6 |
|  |  | 95% CI | 8.4-11.0 | 10.7-10.9 | 10.4-10.5 | 10.6-10.7 | 10.7-10.8 | 10.7-11.2 |
|  |  | Total N | 11 | 599 | 2088 | 8847 | 2557 | 157 |
| % with anemia | Overall | % | 23.8 | 41.8 | 49.2 | 48.6 | 49.4 | 48.7 |
|  |  | 95% CI | 17.0-30.6 | 39.8-43.8 | 47.8-50.7 | 47.8-49.4 | 47.9-51.0 | 42.4-55.1 |
|  |  | Total N | 151 | 2429 | 4617 | 14,838 | 4001 | 238 |
|  | Asia | % | 18.1 | 38.1 | 38.6 | 37.4 | 46.6 | 52.6 |
|  |  | 95% CI | 11.4-24.8 | 35.8-40.4 | 36.7-40.5 | 36.2-38.7 | 43.9-49.3 | 41.4-63.9 |
|  |  | Total N | 127 | 1707 | 2439 | 5478 | 1323 | 76 |
|  | Africa | % | 81.8 | 53.4 | 62.1 | 56.2 | 51.5 | 47.1 |
|  |  | 95% CI | 59.0-100.0 | 49.4-57.4 | 60.0-64.2 | 55.2-57.2 | 49.6-53.5 | 39.3-54.9 |
|  |  | Total N | 11 | 599 | 2088 | 8847 | 2557 | 157 |
| Mean weight (kg) | Overall | Mean | 43.7 | 45.5 | 47.4 | 49.4 | 51.3 | 51.6 |
|  |  | SD | 6.8 | 7.2 | 8.2 | 10.1 | 11.8 | 12.4 |
|  |  | 95% CI | 43.2-44.2 | 45.4-45.6 | 47.3-47.6 | 49.3-49.5 | 51.1-51.6 | 50.6-52.6 |
|  |  | Total N | 683 | 9107 | 13,633 | 47,155 | 11,524 | 570 |
|  | Asia | Mean | 42.0 | 43.5 | 44.5 | 45.5 | 45.6 | 45.8 |
|  |  | SD | 5.3 | 5.4 | 6.1 | 7.4 | 8.4 | 8.4 |
|  |  | 95% CI | 41.6-42.5 | 43.4-43.6 | 44.4-44.6 | 45.4-45.5 | 46.4-46.7 | 44.9-46.7 |
|  |  | Total N | 577 | 7229 | 9689 | 29,702 | 7261 | 331 |
|  | Africa | Mean | 50.7 | 51.9 | 55.2 | 57.5 | 60.3 | 59.7 |
|  |  | SD | 7.1 | 7.0 | 7.8 | 9.8 | 12.0 | 12.2 |
|  |  | 95% CI | 47.5-53.8 | 51.5-52.3 | 54.8-55.5 | 57.3-57.7 | 59.9-60.7 | 58.0-61.3 |
|  |  | Total N | 22 | 1000 | 2589 | 11,300 | 3543 | 220 |
| % underweight | Overall | % | 0.6 | 3.0 | 4.3 | 25.2 | 22.5 | 22.4 |
|  |  | 95% CI | 0.01-1.2 | 2.6-3.3 | 3.9-4.6 | 24.8-25.6 | 21.7-23.2 | 19.0-25.9 |
|  |  | Total N | 679 | 8930 | 13,308 | 46,646 | 11,394 | 566 |
|  | Asia | % | 0.7 | 3.5 | 5.1 | 33.3 | 30.4 | 32.0 |
|  |  | 95% CI | 0.02-1.4 | 3.1-3.9 | 4.6-5.5 | 32.8-33.8 | 29.4-31.5 | 27.0-37.1 |
|  |  | Total N | 575 | 7062 | 9468 | 29,674 | 7255 | 331 |
|  | Africa | % | 0.0 | 0.8 | 1.6 | 6.6 | 7.5 | 8.3 |
|  |  | 95% CI | 0.0-0.0 | 0.3-1.4 | 1.1-2.1 | 6.1-7.0 | 6.6-8.4 | 4.7-12.0 |
|  |  | Total N | 21 | 996 | 2494 | 10,844 | 3421 | 216 |
| % overweight | Overall | % | 9.7 | 4.6 | 6.4 | 10.6 | 16.2 | 17.3 |
|  |  | 95% CI | 7.5-11.9 | 4.1-5.0 | 6.0-6.9 | 10.3-10.9 | 15.5-16.9 | 14.2-20.4 |
|  |  | Total N | 679 | 8930 | 13,308 | 46,646 | 11,394 | 566 |
|  | Asia | % | 4.7 | 2.1 | 2.2 | 3.9 | 7.7 | 7.6 |
|  |  | 95% CI | 3.0-6.4 | 1.8-2.5 | 1.9-2.5 | 3.7-4.1 | 7.1-8.3 | 4.7-10.4 |
|  |  | Total N | 575 | 7062 | 9468 | 29,674 | 7255 | 331 |
|  | Africa | % | 28.6 | 6.8 | 17.5 | 24.6 | 33.0 | 30.1 |
|  |  | 95% CI | 9.3-47.9 | 5.3-8.4 | 16.0-19.0 | 23.8-25.4 | 31.4-34.6 | 24.0-36.2 |
|  |  | Total N | 21 | 996 | 2494 | 10,844 | 3421 | 216 |
| Mean height (cm) | Overall | Mean | 149.6 | 150.8 | 152.0 | 153.6 | 153.9 | 154.0 |
|  |  | SD | 6.1 | 6.3 | 6.5 | 7.0 | 7.1 | 7.5 |
|  |  | 95% CI | 149.1-150.0 | 150.7-151.0 | 151.9-152.1 | 153.5-153.6 | 153.8-154.1 | 153.4-154.6 |
|  |  | Total N | 689 | 9134 | 13,670 | 47,539 | 11,625 | 578 |
|  | Asia | Mean | 148.5 | 149.2 | 150.1 | 151.4 | 151.3 | 150.4 |
|  |  | SD | 5.4 | 5.2 | 5.6 | 6.2 | 6.0 | 5.8 |
|  |  | 95% CI | 148.0-148.9 | 149.1-140.3 | 150.0-150.2 | 151.3-151.5 | 151.2-151.4 | 149.8-151.0 |
|  |  | Total N | 585 | 7244 | 9738 | 30,053 | 7352 | 336 |
|  | Africa | Mean | 156.4 | 157.9 | 156.9 | 157.9 | 158.9 | 159.2 |
|  |  | SD | 5.6 | 6.1 | 6.4 | 6.5 | 6.4 | 6.6 |
|  |  | 95% CI | 153.9-158.9 | 157.6-158.3 | 156.6-157.1 | 157.8-158.0 | 158.7-159.1 | 158.3-160.0 |
|  |  | Total N | 21 | 1015 | 2578 | 11,208 | 3507 | 222 |
| % low stature | Overall | % | 24.2 | 40.4 | 40.8 | 42.1 | 40.7 | 40.5 |
|  |  | 95% CI | 21.0-27.4 | 39.4-41.4 | 39.9-41.6 | 41.6-42.5 | 39.8-41.6 | 36.5-44.5 |
|  |  | Total N | 689 | 9134 | 13,670 | 47,539 | 11,625 | 578 |
|  | Asia | % | 28.0 | 48.8 | 50.5 | 55.5 | 55.9 | 60.7 |
|  |  | 95% CI | 24.4-31.7 | 47.7-50.0 | 49.5-51.5 | 54.9-56.1 | 54.7-57.0 | 55.5-65.9 |
|  |  | Total N | 585 | 7244 | 9738 | 30,053 | 7352 | 336 |
|  | Africa | % | 4.8 | 5.4 | 16.9 | 16.5 | 13.2 | 11.3 |
|  |  | 95% CI | 0.0-13.9 | 4.0-6.8 | 15.5-18.4 | 15.9-17.2 | 12.1-14.4 | 7.1-15.4 |
|  |  | Total N | 21 | 1015 | 2578 | 11,208 | 3507 | 222 |
| Mean # antenatal care visits | Overall | Mean | 0.5 | 0.8 | 1.1 | 1.2 | 1.2 | 1.0 |
|  |  | SD | 1.1 | 1.7 | 1.9 | 1.9 | 1.9 | 1.8 |
|  |  | 95% CI | 0.4-0.5 | 0.8-0.9 | 1.0-1.1 | 1.2-1.2 | 1.2-1.3 | 0.9-1.2 |
|  |  | Total N | 2267 | 14,071 | 13,527 | 44,086 | 10,439 | 522 |
|  | Asia | Mean | 0.5 | 0.7 | 0.9 | 1.0 | 0.8 | 0.4 |
|  |  | SD | 1.0 | 1.5 | 1.8 | 1.7 | 1.5 | 1.0 |
|  |  | 95% CI | 0.4-0.5 | 0.7-0.7 | 0.9-1.0 | 1.0-1.0 | 0.8-0.8 | 0.3-0.5 |
|  |  | Total N | 2254 | 13,392 | 12,772 | 40,760 | 9064 | 414 |
|  | Africa | Mean | 3.9 | 3.6 | 3.6 | 3.9 | 4.0 | 3.6 |
|  |  | SD | 2.0 | 1.8 | 1.6 | 1.7 | 1.7 | 1.9 |
|  |  | 95% CI | 2.6-5.1 | 3.5-3.7 | 3.5-3.7 | 3.8-3.9 | 3.9-4.0 | 3.2-3.9 |
|  |  | Total N | 13 | 679 | 755 | 3326 | 1375 | 108 |
| % with skilled birth assistance | Overall | % | 98.6 | 95.4 | 94.2 | 95.5 | 92.9 | 93.0 |
|  |  | 95% CI | 98.0-99.1 | 95.0-95.7 | 93.8-94.5 | 95.3-95.6 | 92.5-93.4 | 91.2-94.9 |
|  |  | Total N | 2011 | 14,904 | 17,880 | 57,746 | 14,216 | 730 |
|  | Asia | % | 98.6 | 95.4 | 93.9 | 95.3 | 92.3 | 92.4 |
|  |  | 95% CI | 98.1-99.1 | 95.0-95.7 | 93.5-94.2 | 95.1-95.5 | 91.9-92.8 | 90.3-94.5 |
|  |  | Total N | 2003 | 14,585 | 16,311 | 50,813 | 12,226 | 631 |
|  | Africa | % | 87.5 | 97.2 | 97.3 | 96.7 | 96.7 | 97.0 |
|  |  | 95% CI | 64.6-100.0 | 95.4-99.006 | 96.5-98.1 | 96.3-97.1 | 95.9-97.5 | 93.6-100.0 |
|  |  | Total N | 8 | 319 | 1569 | 6933 | 1990 | 99 |

Total N = effective sample size; Unstable estimates in red.

Note: Categorical variables are defined as follows: anemia, % with anemia at enrolment (adolescents: Hb <110g/L; adults Hb <120g/L)); low stature, % with low stature at enrolment (adolescents: ≤-2 SD HAZ; adults <152cm); overweight, % overweight at enrolment (adolescents: ≥+1 SD WAZ; adults: BMI ≥25); underweight, % underweight at enrolment (adolescents: ≤-2 SD WAZ; adults: BMI <18.5).

**Table S7:** Outcomes adjusted for design effect (fixed study effect, intervention given) and adjusted for design effect plus confounders (education, parity).

|  |  |  | 10-14 years | 15-17 years | 18-19 years | 20-29 years | 30-39 years | 40+  years |
| --- | --- | --- | --- | --- | --- | --- | --- | --- |
| OUTCOMES ADJUSTED BY DESIGN EFFECT | | | | | | | | |
| Mean (SE) gestational age (weeks) | Overall | Mean | 38.47 | 38.70 | 38.76 | 38.80 | 38.45 | 38.25 |
|  |  | SE | 0.06 | 0.03 | 0.02 | 0.02 | 0.03 | 0.10 |
|  |  | 95% CI | 38.35-38.60 | 38.64-38.75 | 38.71-38.81 | 38.76-38.83 | 38.39-38.50 | 38.05-38.46 |
|  |  | Total N | 2389 | 17,568 | 20,840 | 69,063 | 16,465 | 832 |
|  | Asia | Mean | 38.37 | 38.61 | 38.70 | 38.67 | 38.16 | 37.92 |
|  |  | SE | 0.07 | 0.03 | 0.03 | 0.02 | 0.03 | 0.12 |
|  |  | 95% CI | 38.24-38.50 | 38.55-38.67 | 38.65-38.76 | 38.63-38.71 | 38.10-38.22 | 37.69-38.16 |
|  |  | Total N | 2293 | 15,904 | 17,195 | 52,612 | 12,556 | 634 |
|  | Africa | Mean | 36.72 | 38.14 | 38.34 | 38.82 | 38.99 | 38.88 |
|  |  | SE | 0.70 | 0.12 | 0.09 | 0.07 | 0.08 | 0.23 |
|  |  | 95% CI | 35.36-38.09 | 37.90-38.37 | 38.17-38.51 | 38.68-38.96 | 38.82-39.15 | 38.42-39.34 |
|  |  | Total N | 19 | 841 | 2380 | 10,509 | 3176 | 180 |
| Mean (SE) birthweight (grams) | Overall | Mean | 2728.37 | 2790.07 | 2841.76 | 2921.42 | 2948.29 | 2918.92 |
|  |  | SE | 14.06 | 5.33 | 4.62 | 2.99 | 4.76 | 20.05 |
|  |  | 95% CI | 2700.82-2755.93 | 2779.62-2800.51 | 2932.71-2850.82 | 2915.56-2927.28 | 2938.97-2957.62 | 2879.62-2958.21 |
|  |  | Total N | 1072 | 8912 | 11,877 | 45,159 | 10,783 | 503 |
|  | Asia | Mean | 2588.90 | 2650.59 | 2711.09 | 2799.25 | 2805.15 | 2760.35 |
|  |  | SE | 13.97 | 5.60 | 5.11 | 3.43 | 5.58 | 23.43 |
|  |  | 95% CI | 2561.52-2616.28 | 2639.61-2661.56 | 2701.07-2721.10 | 2792.53-2805.97 | 2794.22-2816.08 | 2714.43-2806.28 |
|  |  | Total N | 976 | 7316 | 8374 | 29,233 | 7027 | 329 |
|  | Africa | Mean | 2618.53 | 2789.25 | 2886.06 | 3011.14 | 3075.03 | 3076.67 |
|  |  | SE | 109.58 | 20.26 | 15.03 | 12.27 | 14.16 | 39.78 |
|  |  | 95% CI | 2403.74-2833.33 | 2749.55-2828.96 | 2856.60-2915.52 | 2987.09-3035.19 | 3047.27-3102.78 | 2998.70-3154.64 |
|  |  | Total N | 19 | 772 | 2247 | 10,005 | 3025 | 156 |
| % anemic (Hb <11.0 g/dL in third trimester) | Overall | % | 34.88 | 38.01 | 36.28 | 33.28 | 34.14 | 37.01 |
|  |  | 95% CI | 27.10-43.55 | 35.95-40.12 | 34.77-37.82 | 32.36-34.21 | 32.67-35.61 | 31.40-42.99 |
|  |  | Total N | 169 | 2670 | 5121 | 17,681 | 5183 | 292 |
|  | Asia | % | 30.62 | 34.35 | 31.12 | 30.67 | 34.60 | 44.08 |
|  |  | 95% CI | 22.51-40.13 | 31.96-36.82 | 29.23-33.08 | 29.50-31.87 | 32.55-36.71 | 35.40-53.14 |
|  |  | Total N | 143 | 1934 | 2910 | 8197 | 2461 | 128 |
|  | Africa | % | 62.43 | 38.25 | 37.66 | 32.35 | 30.68 | 28.71 |
|  |  | 95% CI | 32.19-85.34 | 33.64-43.07 | 34.19-41.25 | 29.62-35.22 | 27.67-33.86 | 21.91-36.63 |
|  |  | Total N | 12 | 603 | 2103 | 8887 | 2569 | 159 |
| % preterm births (<37 weeks) | Overall | % | 16.95 | 15.11 | 14.56 | 14.16 | 16.73 | 17.9 |
|  |  | 95% CI | 15.89-18.09 | 14.54-15.71 | 14.02-15.12 | 13.72-14.62 | 16.11-17.37 | 16.19-19.80 |
|  |  | Total N | 2565 | 19,101 | 22,874 | 76,537 | 18,677 | 995 |
|  | Asia | % | 19.04 | 16.92 | 16.07 | 16.11 | 20.16 | 21.6 |
|  |  | 95% CI | 17.83-20.34 | 16.25-17.61 | 15.45-16.72 | 15.58-16.66 | 19.37-20.98 | 19.32-24.15 |
|  |  | Total N | 2459 | 17,196 | 18,815 | 58,481 | 14,251 | 749 |
|  | Africa | % | 23.94 | 18.84 | 18.71 | 14.86 | 13.74 | 15.2 |
|  |  | 95% CI | 17.02-33.67 | 16.49-21.52 | 16.54-21.17 | 13.33-16.57 | 12.17-15.53 | 11.72-19.71 |
|  |  | Total N | 22 | 1023 | 2694 | 11,753 | 3658 | 226 |
| % low birthweight (<2500 grams) | Overall | % | 20.11 | 18.83 | 16.89 | 13.72 | 13.99 | 14.76 |
|  |  | 95% CI | 19.01-21.27 | 18.12-19.58 | 16.25-17.56 | 13.25-14.20 | 13.41-14.60 | 12.99-16.77 |
|  |  | Total N | 1072 | 8912 | 11,877 | 45,159 | 10,783 | 503 |
|  | Asia | % | 29.96 | 28.09 | 25.11 | 20.45 | 21.18 | 22.34 |
|  |  | 95% CI | 28.29-31.74 | 26.97-29.24 | 24.11-26.15 | 19.71-21.22 | 20.25-22.16 | 19.60-25.47 |
|  |  | Total N | 1072 | 8912 | 11,877 | 45,159 | 10,783 | 503 |
|  | Africa | % | 41.2 | 17.99 | 12.3 | 7.8 | 7.09 | 7.26 |
|  |  | 95% CI | 24.90-68.18 | 14.40-22.47 | 10.05-15.06 | 6.51-9.35 | 5.75-8.76 | 4.12-12.79 |
|  |  | Total N | 19 | 772 | 2247 | 10,005 | 3025 | 156 |
| % small-for-gestational-age (<10^th^ centile) | Overall | % | 29.3 | 27.72 | 25.58 | 21.95 | 18.96 | 20.37 |
|  |  | 95% CI | 27.73-30.95 | 26.84-28.63 | 24.78-26.41 | 21.36-22.56 | 18.27-19.67 | 18.03-23.02 |
|  |  | Total N | 1072 | 8912 | 11,879 | 45,165 | 10,785 | 503 |
|  | Asia | % | 41.8 | 39.6 | 36.46 | 30.98 | 27.18 | 28.89 |
|  |  | 95% CI | 39.57-44.16 | 38.35-40.89 | 35.32-37.65 | 30.13-31.86 | 26.15-28.25 | 25.37-32.90 |
|  |  | Total N | 1072 | 8912 | 11,879 | 45,165 | 10,785 | 503 |
|  | Africa | % | 33.23 | 24.03 | 20.26 | 16.33 | 12.93 | 13.63 |
|  |  | 95% CI | 17.11-64.53 | 20.28-28.47 | 17.61-23.31 | 14.46-18.45 | 11.17-14.96 | 9.03-20.57 |
|  |  | Total N | 19 | 772 | 2249 | 10,006 | 3026 | 156 |
| % stillbirth | Overall | % | 3.07 | 2.85 | 2.6 | 2.37 | 3.11 | 4.49 |
|  |  | 95% CI | 2.52-3.74 | 2.57-3.16 | 2.35-2.87 | 2.19-2.57 | 2.82-3.44 | 3.46-5.81 |
|  |  | Total N | 2544 | 18,726 | 22,295 | 73,940 | 17,820 | 933 |
|  | Asia | % | 3.8 | 3.54 | 3.19 | 2.92 | 3.89 | 5.06 |
|  |  | 95% CI | 3.12-4.62 | 3.21-3.92 | 2.89-3.52 | 2.70-3.16 | 3.52-4.30 | 3.76-6.81 |
|  |  | Total N | 2445 | 16,983 | 18,450 | 56,688 | 13,663 | 710 |
|  | Africa | % | / | 3.58 | 2.96 | 2.52 | 3.33 | 6.64 |
|  |  | 95% CI | / | 2.15-5.95 | 1.93-4.55 | 1.72-3.71 | 2.22-5.00 | 3.57-12.35 |
|  |  | Total N | 20 | 910 | 2555 | 11,187 | 3414 | 204 |
| % perinatal mortality | Overall | % | 3.92 | 2.77 | 2.42 | 1.62 | 1.91 | 2.16 |
|  |  | 95% CI | 3.28-4.67 | 2.48-3.10 | 2.17-2.71 | 1.47-1.79 | 1.69-2.17 | 1.46-3.20 |
|  |  | Total N | 2514 | 18,177 | 20,467 | 65,867 | 15,552 | 822 |
|  | Asia | % | 4.75 | 3.32 | 2.91 | 1.96 | 2.28 | 2.47 |
|  |  | 95% CI | 4.00-5.65 | 2.98-3.70 | 2.61-3.23 | 1.79-2.15 | 2.01-2.58 | 1.63-3.76 |
|  |  | Total N | 2445 | 16,983 | 18,448 | 56,685 | 13,663 | 710 |
|  | Africa | % | / | 3.31 | 2.67 | 1.2 | 1.93 | 2.17 |
|  |  | 95% CI | / | 1.82-6.01 | 1.54-4.62 | 0.74-1.93 | 1.13-3.30 | 0.53-8.88 |
|  |  | Total N | 9 | 512 | 855 | 3768 | 1306 | 98 |
| % neonatal mortality | Overall | % | 5.02 | 3.71 | 3.27 | 2.2 | 2.53 | 2.93 |
|  |  | 95% CI | 4.30-5.87 | 3.37-4.08 | 2.97-3.59 | 2.03-2.38 | 2.28-2.82 | 2.10-4.10 |
|  |  | Total N | 2400 | 17,465 | 19,761 | 63,709 | 14,873 | 771 |
|  | Asia | % | 6.18 | 4.5 | 3.97 | 2.69 | 3.07 | 3.52 |
|  |  | 95% CI | 5.30-7.19 | 4.11-4.94 | 3.63-4.35 | 2.49-2.91 | 2.76-3.42 | 2.48-4.99 |
|  |  | Total N | 2333 | 16,292 | 17,789 | 54,725 | 13,034 | 667 |
|  | Africa | % | / | 4.56 | 3.54 | 1.74 | 2.65 | 2.35 |
|  |  | 95% CI | / | 2.73-7.63 | 2.20-5.69 | 1.17-2.60 | 1.67-4.20 | 0.58-9.50 |
|  |  | Total N | 9 | 496 | 830 | 3684 | 1264 | 91 |
| OUTCOMES ADJUSTED FOR DESIGN EFFECT AND CONFOUNDERS | | | | | | | | |
| Mean (SE) gestational age (weeks) | Overall | Mean | 38.30 | 38.53 | 38.61 | 38.70 | 38.36 | 38.20 |
|  |  | SE | 0.07 | 0.03 | 0.03 | 0.02 | 0.03 | 0.11 |
|  |  | 95% CI | 38.17-38.43 | 38.47-38.59 | 38.56-38.67 | 38.66-38.73 | 38.31-38.42 | 37.98-38.42 |
|  |  | Total N | 2389 | 17,568 | 20,840 | 69,063 | 16,465 | 832 |
|  | Asia | Mean | 38.28 | 38.53 | 38.64 | 38.67 | 38.21 | 37.99 |
|  |  | SE | 0.07 | 0.03 | 0.03 | 0.02 | 0.03 | 0.13 |
|  |  | 95% CI | 38.15-38.42 | 38.47-38.59 | 38.58-38.70 | 38.63-38.71 | 38.15-38.27 | 37.75-38.24 |
|  |  | Total N | 2293 | 15,904 | 17,195 | 52,612 | 12,556 | 634 |
|  | Africa | Mean | 36.66 | 37.62 | 37.96 | 38.53 | 38.79 | 38.75 |
|  |  | SE | 1.03 | 0.21 | 0.18 | 0.16 | 0.17 | 0.30 |
|  |  | 95% CI | 34.64-38.69 | 37.21-38.03 | 37.62-38.30 | 38.22-38.85 | 38.46-39.12 | 38.17-39.33 |
|  |  | Total N | 19 | 841 | 2380 | 10,509 | 3176 | 180 |
| Mean (SE) birthweight (grams) | Overall | Mean | 2720.44 | 2770.81 | 2810.47 | 2869.71 | 2880.15 | 2851.94 |
|  |  | SE | 14.51 | 6.02 | 5.03 | 3.25 | 5.26 | 20.70 |
|  |  | 95% CI | 2692.00-2748.88 | 2759.01-2782.61 | 2800.61-2820.34 | 2863.34-2876.08 | 2869.84-2890.47 | 2811.38-2892.50 |
|  |  | Total N | 1072 | 8912 | 11,877 | 45,159 | 10,783 | 503 |
|  | Asia | Mean | 2638.47 | 2691.05 | 2735.07 | 2797.02 | 2800.48 | 2755.87 |
|  |  | SE | 14.26 | 6.16 | 5.36 | 3.51 | 5.94 | 24.21 |
|  |  | 95% CI | 2610.53-2666.41 | 2678.98-2703.12 | 2724.56-2745.59 | 2790.14-2803.90 | 2788.84-2812.12 | 2708.41-2803.33 |
|  |  | Total N | 976 | 7316 | 8374 | 29,233 | 7027 | 329 |
|  | Africa | Mean | 2513.66 | 2813.07 | 2865.68 | 2917.14 | 2939.90 | 2940.97 |
|  |  | SE | 153.58 | 34.03 | 28.56 | 26.63 | 27.81 | 48.37 |
|  |  | 95% CI | 2212.62-2814.70 | 2746.37-2879.77 | 2809.69-2921.67 | 2864.94-2969.34 | 2885.39-2994.41 | 2846.15-3035.79 |
|  |  | Total N | 19 | 772 | 2247 | 10,005 | 3025 | 156 |
| % anemic (Hb <11.0 g/dL in third trimester) | Overall | % | 36.32 | 37.87 | 35.40 | 31.70 | 31.49 | 33.65 |
|  |  | 95% CI | 27.50-46.18 | 35.41-40.40 | 33.65-37.20 | 30.64-32.79 | 29.77-33.26 | 27.49-40.43 |
|  |  | Total N | 169 | 2670 | 5121 | 17,681 | 5183 | 292 |
|  | Asia | % | 34.40 | 36.76 | 33.54 | 29.78 | 31.15 | 40.72 |
|  |  | 95% CI | 25.59-44.44 | 34.13-39.47 | 31.43-35.71 | 28.56-31.02 | 28.92-33.47 | 31.29-50.89 |
|  |  | Total N | 143 | 1934 | 2910 | 8197 | 2461 | 128 |
|  | Africa | % | / | 32.39 | 30.87 | 28.06 | 26.70 | 23.10 |
|  |  | 95% CI | / | 24.48-41.45 | 24.45-38.13 | 22.34-34.58 | 20.97-33.34 | 15.41-33.13 |
|  |  | Total N | 12 | 603 | 2103 | 8887 | 2569 | 159 |
| % preterm births (<37 weeks) | Overall | % | 23.1 | 20.62 | 19.76 | 18.82 | 21.73 | 22.95 |
|  |  | 95% CI | 21.59-24.70 | 19.77-21.50 | 18.95-20.61 | 18.07-19.61 | 20.74-22.77 | 20.65-25.51 |
|  |  | Total N | 2565 | 19,101 | 22,874 | 76,537 | 18,677 | 995 |
|  | Asia | % | 23.14 | 20.52 | 19.41 | 18.94 | 22.9 | 24.3 |
|  |  | 95% CI | 21.60-24.80 | 19.62-21.46 | 18.55-20.32 | 18.12-19.80 | 21.77-24.08 | 21.64-27.29 |
|  |  | Total N | 2459 | 17,196 | 18,815 | 58,481 | 14,251 | 749 |
|  | Africa | % | 25.75 | 22.64 | 23.25 | 18.16 | 16.6 | 17.87 |
|  |  | 95% CI | 15.06-44.02 | 18.87-27.17 | 19.48-27.75 | 15.28-21.60 | 13.77-20.01 | 13.11-24.36 |
|  |  | Total N | 22 | 1023 | 2694 | 11,753 | 3658 | 226 |
| % low birthweight (<2500 grams) | Overall | % | 25.93 | 24.88 | 23.28 | 20.27 | 20.91 | 22.04 |
|  |  | 95% CI | 24.49-27.45 | 23.87-25.93 | 22.32-24.28 | 19.44-21.14 | 19.90-21.98 | 19.35-25.09 |
|  |  | Total N | 1072 | 8912 | 11,877 | 45,159 | 10,783 | 503 |
|  | Asia | % | 33.08 | 31.7 | 29.53 | 25.74 | 26.6 | 27.96 |
|  |  | 95% CI | 31.18-35.10 | 30.33-33.12 | 28.24-30.89 | 24.62-26.92 | 25.24-28.04 | 24.46-31.96 |
|  |  | Total N | 976 | 7316 | 8374 | 29,233 | 7027 | 329 |
|  | Africa | % | 79.65 | 28.46 | 25.2 | 22.76 | 23.93 | 25.73 |
|  |  | 95% CI | 50.09-126.64 | 22.38-36.18 | 20.29-31.28 | 18.37-28.19 | 18.98-30.17 | 17.40-38.04 |
|  |  | Total N | 19 | 772 | 2247 | 10,005 | 3025 | 156 |
| % small-for-gestational-age (<10^th^ centile) | Overall | % | 35.22 | 33.86 | 32.12 | 28.78 | 25.29 | 27.54 |
|  |  | 95% CI | 33.28-37.27 | 32.68-35.07 | 31.01-33.27 | 27.81-29.79 | 24.21-26.42 | 24.22-31.32 |
|  |  | Total N | 1072 | 8912 | 11,879 | 45,165 | 10,785 | 503 |
|  | Asia | % | 42.68 | 40.98 | 38.7 | 34.41 | 30.39 | 33.28 |
|  |  | 95% CI | 40.29-45.21 | 39.51-42.50 | 37.30-40.14 | 33.19-35.66 | 29.03-31.81 | 29.12-38.03 |
|  |  | Total N | 976 | 7316 | 8374 | 29,238 | 7028 | 329 |
|  | Africa | % | 53.04 | 20.69 | 20.69 | 20.7 | 18.5 | 19.19 |
|  |  | 95% CI | 24.20-116.25 | 15.51-27.60 | 16.03-26.71 | 16.08-26.64 | 14.05-24.37 | 11.68-31.53 |
|  |  | Total N | 19 | 772 | 2249 | 10,006 | 3026 | 156 |
| % stillbirth | Overall | % | 4.16 | 4.05 | 3.99 | 4.53 | 6.5 | 9.2 |
|  |  | 95% CI | 3.40-5.10 | 3.62-4.53 | 3.58-4.45 | 4.10-5.02 | 5.75-7.35 | 6.99-12.12 |
|  |  | Total N | 2544 | 18,726 | 22,295 | 73,940 | 17,820 | 933 |
|  | Asia | % | 4.43 | 4.27 | 4.24 | 4.8 | 6.84 | 8.89 |
|  |  | 95% CI | 3.61-5.44 | 3.80-4.80 | 3.78-4.75 | 4.32-5.35 | 6.00-7.79 | 6.52-12.13 |
|  |  | Total N | 2445 | 16,983 | 18,450 | 56,688 | 13,663 | 710 |
|  | Africa | % | / | / | / | / | / | / |
|  |  | 95% CI | / | / | / | / | / | / |
|  |  | Total N | 20 | 910 | 2555 | 11,187 | 3414 | 204 |
| % perinatal mortality | Overall | % | 4.59 | 3.38 | 3.3 | 2.86 | 3.59 | 3.91 |
|  |  | 95% CI | 3.79-5.57 | 2.95-3.87 | 2.88-3.78 | 2.51-3.27 | 3.05-4.23 | 2.54-6.00 |
|  |  | Total N | 2514 | 18,177 | 20,467 | 65,867 | 15,552 | 822 |
|  | Asia | % | 5.57 | 4.07 | 3.99 | 3.48 | 4.32 | 4.84 |
|  |  | 95% CI | 4.63-6.69 | 3.59-4.61 | 3.52-4.51 | 3.08-3.93 | 3.70-5.05 | 3.16-7.42 |
|  |  | Total N | 2445 | 16,983 | 18,448 | 56,685 | 13,663 | 710 |
|  | Africa | % | / | / | / | / | / | / |
|  |  | 95% CI | / | / | / | / | / | / |
|  |  | Total N | 9 | 512 | 855 | 3768 | 1306 | 98 |
| % neonatal mortality | Overall | % | 6.25 | 4.78 | 4.61 | 3.84 | 4.57 | 5.25 |
|  |  | 95% CI | 5.29-7.39 | 4.27-5.36 | 4.11-5.17 | 3.43-4.30 | 3.97-5.25 | 3.66-7.52 |
|  |  | Total N | 2400 | 17,465 | 19,761 | 63,709 | 14,873 | 771 |
|  | Asia | % | 7.44 | 5.66 | 5.47 | 4.57 | 5.38 | 6.4 |
|  |  | 95% CI | 6.33-8.76 | 5.09-6.30 | 4.92-6.09 | 4.12-5.08 | 4.70-6.15 | 4.48-9.16 |
|  |  | Total N | 2333 | 16,292 | 17,789 | 54,725 | 13,034 | 667 |
|  | Africa | % | / | / | / | / | / | / |
|  |  | 95% CI | / | / | / | / | / | / |
|  |  | Total N | 9 | 496 | 830 | 3684 | 1264 | 91 |

Total N = effective sample size; Unstable estimates in red; / = model did not converge.

**Figure S1:** Region-specific preterm birth (%) stratified by maternal age group.

**Figure S2:** Region-specific low birthweight (LBW) (%) stratified by maternal age group.

**Figure S3:** Region-specific small-for-gestational age (SGA) (%) stratified by maternal age group.

**Figure S4:** Region-specific stillbirths (%) stratified by maternal age group.

**Figure S5:** Region-specific neonatal mortality (%) stratified by maternal age group.

**Figure S6:** Perinatal mortality (%) stratified by maternal age.

**Figure S7:** Region-specific perinatal mortality (%) stratified by maternal age group.

**Figure S8:** Region-specific anemia (%) stratified by maternal age group.

**Appendix 2: Risk of Bias Assessment for Individual Studies**

**Adu-Afarwuah^1^:**

| **Bias** | **Authors’ Judgement** | **Support for Judgement** |
| --- | --- | --- |
| Random sequence generation (selection bias) | Low risk | Quote: "women were randomly allocated into one of 3 groups by using a computer-generated scheme (SAS version 9.3; SAS Institute) in blocks of 9."  Comment: probably done |
| Allocation concealment (selection bias) | Low risk | Quote: "Sheets bearing supplement allocations represented by 6 different color codes (3 for IFA and 3 for MMN) and an inscription “LNS” (for the LNS group) and numbered 1–1320 were placed in opaque envelopes and stacked in increasing order. At each enrolment, the study nurse shuffled the 9 topmost envelopes in the stack, and the woman picked one to reveal allocation. Allocation information was kept by the field supervisor (HO) in a password-protected file, which was shared with the study statistician (JMP) at UC Davis, who designed the randomisation scheme."  Comment: probably done |
| Blinding of participants and personnel (performance bias) | Low risk | Quote: "Two individuals in Ghana who were independent of the research team color-coded the capsules by placing color stickers (which also included the letter P or L to indicate pregnancy or lactation) on the blister packs of IFA and MMN, so that no investigator, study worker, or participant knew the identities of the capsules except by the colors."  Comment: participants and caregivers were probably blinded to the treatment assignment. |
| Blinding of outcome assessment (detection bias) | Low risk | Quote: "none of the maternal or newborn anthropometrists was aware of the code allocations. Likewise, data analysts remained blinded until all preliminary analyses had been completed, and the allocation codes were broken."  Comment: outcome assessors were probably blinded to the treatment assignment. |
| Incomplete outcome data (attrition bias) | Low risk | Exclusion (until delivery) was 20% (and was balanced between treatment arms); the reason was reported. Attrition (until delivery) was 4.4% and reasons were reported |
| Selective reporting (reporting bias) | Low risk | Comment: results of all outcomes mentioned in methods section were presented in the paper |
| Other bias | Low risk | Comment: no other bias was identified |

**Ashorn^2^:**

| **Bias** | **Authors’ Judgement** | **Support for Judgement** |
| --- | --- | --- |
| Random sequence generation (selection bias) | Low risk | Quote: "A study statistician not involved in data collection generated 4 randomization code lists in blocks of 9 (one list for each of the 4 enrolment sites)."  Comment: probably done |
| Allocation concealment (selection bias) | Low risk | Quote: "a researcher not involved with the trial created individual randomisation slips (in blocks of 9) and packed them in sealed, numbered, opaque randomisation envelopes that were stored in numerical order. Eligible pregnant women were requested to choose 1 of the top 6 envelopes in the stack, and the contents of the envelope indicated her participant number and group allocation."  Comment: probably done |
| Blinding of participants and personnel (performance bias) | Low risk | Quote: "The IFA and MMN interventions were provided by using double-masked procedures—that is, the capsules looked identical, and neither the participants nor the research team members were aware of the nutrient contents of the supplement capsules."  Comment: participants and caregivers were probably blinded to the treatment assignment. |
| Blinding of outcome assessment (detection bias) | Low risk | Quote: "The data collectors who performed the anthropometric measurements or assessed other outcomes were not aware of group allocation. Researchers responsible for the data cleaning remained blind to the trial code until the database was fully cleaned."  Comment: outcome assessors were probably blinded to the treatment assignment. |
| Incomplete outcome data (attrition bias) | Low risk | Attrition (until delivery) was 6.0% (and was balanced between treatment arms); reasons were not reported |
| Selective reporting (reporting bias) | Low risk | Comment: all outcomes presented in the methods section were reported in the paper. |
| Other bias | Low risk | Comment: no other bias was identified. |

**Belizan^3^:**

| **Bias** | **Authors’ Judgement** | **Support for Judgement** |
| --- | --- | --- |
| Random sequence generation (selection bias) | Low risk | Quote: “They were randomized at each hospital by a random-sample generator program provided by the Epistat Statistical Package.” |
| Allocation concealment (selection bias) | Low risk | Quote: “A complete set of numbered, sealed, opaque envelopes containing the randomization codes was sent to each of the three hospitals. The series of bottles (one bottle for each scheduled prenatal visit) containing all the tablets needed for the entire pregnancy were kept at the central unit. When a woman was enrolled and the corresponding envelope was opened at the hospital, the study number was revealed to the central unit.” |
| Blinding of participants and personnel (performance bias) | Low risk | See above (allocation concealment).  Quote: “Thus, the woman, the nurses, and the physicians responsible for prenatal care were all unaware of the woman's treatment status.”  Quote: “The placebo tablets contained lactose and granulated starch and were identical to the calcium tablets with respect to weight, size, flavor, and color.” |
| Blinding of outcome assessment (detection bias) | Low risk | See above (blinding of participants and personnel).  Quote: “The nurses and physicians were also responsible for distributing the bottles of medications, taking blood-pressure measurements, and collecting blood and urine samples at the follow-up visits, which were scheduled at 23, 25, 27, 31, and 35 weeks and then weekly until delivery.” |
| Incomplete outcome data (attrition bias) | Low risk | Quote: “Twenty-seven women (2.3 percent) were lost to follow-up after randomization (13 in the placebo group and 14 in the calcium group) but before they started any treatment, and therefore were not included in the follow-up analyses. Follow-up was incomplete for 46 women in the placebo group and 52 women in the calcium group because of a change of hospital, physician, or residence. Nonetheless, all were included in the analyses up to the time when they were lost to follow-up.” |
| Selective reporting (reporting bias) | Low risk | Comment: All outcomes were reported on. |
| Other bias | Low risk | Comment: no other bias was identified. |

**Bhutta^4^:**

| **Bias** | **Authors’ Judgement** | **Support for Judgement** |
| --- | --- | --- |
| Random sequence generation (selection bias) | Low risk | Quote: "a cluster-based allocation strategy of supplements (either IF or MMN supplementation) by respective CHWs was implemented".  Comment: probably done |
| Allocation concealment (selection bias) | Low risk | Comment: "allocated to either the IF or MMN supplements according to their respective location and allocation by the AKU Pharmacy".  Comment: probably done |
| Blinding of participants and personnel (performance bias) | Low risk | Quote: "Both tablets were identical in colour, shape and packaging" and "field staff (medical officers, CHWs, social scientists and data collection team) remained completely blinded as to the supplements allocation. All pregnant women were allocated a unique code and allocated a uniquely labelled and numerically coded specific supplement supply". Comment: participants and caregivers were probably blinded to the treatment assignment. |
| Blinding of outcome assessment (detection bias) | Low risk | Quote: "Both tablets were identical in colour, shape and packaging" and "field staff (medical officers, CHWs, social scientists and data collection team) remained completely blinded as to the supplements allocation".  Comment: outcome assessors were probably blinded to the treatment assignment. |
| Incomplete outcome data (attrition bias) | Low risk | Attrition (15.8%) and exclusion (around 1%) along with their reasons were reported. Attrition and exclusions were balanced across the treatment arms. |
| Selective reporting (reporting bias) | Low risk | Comment: results of all outcomes mentioned in methods section were presented in the paper |
| Other bias | Low risk | Comment: no other bias was identified, including cluster-design specific biases (recruitment bias, baseline imbalance, loss of clusters, incorrect analysis, and comparability with individually randomised trials) |

**Christian^5^:**

| **Bias** | **Authors’ Judgement** | **Support for Judgement** |
| --- | --- | --- |
| Random sequence generation (selection bias) | Low risk | Quote: "Randomisation was done in blocks of five within each village development community by the senior study investigators, who drew numbered chips from a hat"  Comment: probably done |
| Allocation concealment (selection bias) | Unclear risk | Quote: "Randomisation was done in blocks of five within each village development community by the senior study investigators, who drew numbered chips from a hat"  Comment: insufficient information to permit judgement |
| Blinding of participants and personnel (performance bias) | Low risk | Quote: "participants, investigators, field staff and statisticians did not know supplement codes", "supplements, which were of identical shape, size, and color" and "code allocation was kept locked at the Johns Hopkins University, Baltimore". Comment: participants and caregivers were blinded to the treatment assignment. |
| Blinding of outcome assessment (detection bias) | Low risk | Quote: "participants, investigators, field staff and statisticians did not know supplement codes" Comment: outcome assessors were blinded to the treatment assignment. |
| Incomplete outcome data (attrition bias) | Low risk | Exclusion (1.43%) and attrition (6.9%) were reported along with their reasons |
| Selective reporting (reporting bias) | Low risk | Comment: results of all outcomes mentioned in methods were presented in the various publications of this trial. |
| Other bias | Low risk | Comment: no other bias was identified, including cluster-design specific biases (recruitment bias, baseline imbalance, loss of clusters, incorrect analysis, and comparability with individually randomised trials). |

**Dewey^6^:**

| **Bias** | **Authors’ Judgement** | **Support for Judgement** |
| --- | --- | --- |
| Random sequence generation (selection bias) | Low risk | Quote: “The final randomization to the 4 arms was then chosen at random from the acceptable potential randomizations; and the letters A, B, C, and D were assigned to the 4 sets, randomly permuting them by sorting on a randomly generated, uniformly distributed number (with the use of SAS for Windows, release 9.2; SAS Institute).” |
| Allocation concealment (selection bias) | Low risk | Quotes: “The trial was designed as a researcher-blind, longitudinal, cluster-randomized effectiveness trial with 4 arms in a ratio of 1:1:1:1.” and “The study evaluation staff received the randomization plan coded only as “A,” “B,” “C,” and “D.” None of the evaluation staff members was involved in supplement delivery.” |
| Blinding of participants and personnel (performance bias) | High risk | Comment: though LNS differs MMN in terms of appearance, the cluster randomization scheme would help to minimize knowledge of participants regarding the other intervention type. However, personnel (home visit teams) may have seen the supplement type when visiting the home. |
| Blinding of outcome assessment (detection bias) | Low risk | Quote: “To the extent possible, both study evaluation teams were kept blind to group assignment, although this was difficult for home visit team members because they might have seen supplements in the home. The distribution of the supplements was coordinated and implemented by LAMB staff, and study evaluation staff only knew group assignment by the prescribed letter (A–D) as described previously.” |
| Incomplete outcome data (attrition bias) | Low risk | Comment: low rate of attrition that was mostly due to travel as opposed to non-participation. |
| Selective reporting (reporting bias) | Low risk | Comment: all outcomes mentioned in the methods section were presented in the paper. |
| Other bias | Low risk | LNS distribution was interrupted from 8 August to 20 October 2012, during which, women received only IFA. However, exploratory analyses were conducted to determine the effects of this disruption. |

**Fawzi^7^:**

| **Bias** | **Authors’ Judgement** | **Support for Judgement** |
| --- | --- | --- |
| Random sequence generation (selection bias) | Low risk | Quote: "A list was prepared according to a randomisation sequence in blocks of 20; at enrolment, each eligible women was assigned to the next numbered bottle" and computerised random number generator was used (personal communication)  Comment: probably done |
| Allocation concealment (selection bias) | Low risk | Quote: "Each eligible women was assigned to the next numbered bottle"  Comment: probably done |
| Blinding of participants and personnel (performance bias) | Low risk | Quote: "Active tablets and placebo were similar in shape, size and color and were packaged in identical coded bottles" and "Each eligible women was assigned to the next numbered bottle" Comment: participants and caregivers were blinded to the treatment assignment. |
| Blinding of outcome assessment (detection bias) | Low risk | Quote: "research assistants who assessed the study outcome were unaware of the intervention group"  Comment: outcome assessors were blinded. |
| Incomplete outcome data (attrition bias) | Low risk | Exclusion (0.5%) and attrition (5.4%) were reported with reasons in each arm. |
| Selective reporting (reporting bias) | Low risk | Comment: all outcomes mentioned in the methods section were presented in the paper. |
| Other bias | Low risk | Comment: no other bias was identified. |

**Friis^8^:**

| **Bias** | **Authors’ Judgement** | **Support for Judgement** |
| --- | --- | --- |
| Random sequence generation (selection bias) | Low risk | Quote: "Allocation to daily supplementation with multimicronutrient or identical-looking placebo tablets was based on simple blocked randomisation. The digits 0–5 in a computer-generated random sequence were replaced by 6 preassigned permuted blocks of 4: AABB, ABAB, ABBA, BABA, BBAA, and BAAB; the digits 6–9 were deleted".  Comment: probably done |
| Allocation concealment (selection bias) | Low risk | Quote: "Containers with 110 multimicronutrient or placebo tablets, which were coded A or B, respectively, were delivered by the manufacturer together with the code in 2 sealed envelopes. Duplicate containers, which corresponded to the random sequence, were consecutively numbered from 1 to 1800. The study participants were numbered consecutively at recruitment".  Comment: probably done |
| Blinding of participants and personnel (performance bias) | Low risk | Quote: "double blind", "multimicronutrient or identical-looking placebo tablets" Comment: study participants and care providers were probably blinded to the treatment assignment. |
| Blinding of outcome assessment (detection bias) | Low risk | Quote: "double blind", "multimicronutrient or identical-looking placebo tablets"  Comment: investigators were probably blinded to the treatment assignment. |
| Incomplete outcome data (attrition bias) | High risk | Attrition was > 20% and reasons for it were reported. Exclusions were not reported in the trial |
| Selective reporting (reporting bias) | Low risk | Comment: all outcomes in the methods section were presented in the paper. |
| Other bias | Low risk | Comment: no other bias was identified. |

**Huybregts^9^:**

| **Bias** | **Authors’ Judgement** | **Support for Judgement** |
| --- | --- | --- |
| Random sequence generation (selection bias) | Low risk | Quote: “A randomization scheme was generated by a computer program in permuted blocks of 4.” |
| Allocation concealment (selection bias) | Low risk | Quote: “Randomization numbers were sealed in opaque envelopes by administrative staff. After identifying an eligible subject, the consulting physician in the field opened the next sealed envelope. For allocations to the MMN group, he transmitted the randomization number to a pharmacist packaging the MMN in individual plastic zip sachets containing 31 tablets. For allocation to the FFS group, project staff prepared a plastic bag containing 31 FFS sachets.” |
| Blinding of participants and personnel (performance bias) | High risk | Quote: “Each sachet of MMN or FFS was labeled with the woman’s name, geographic location, and identification number. Home visitors kept both the MMN and FFS with them and visited 10–20 participants per day to provide  and directly observe the supplement intake.”  Comment: women were provided either a fortified spread (LNS) or a tablet (MMN), so differences between groups would have been apparent. Project staff prepared the LNS sachets with participants’ names, geographic locations, and identification numbers on them. Trial was labeled as nonblinded. |
| Blinding of outcome assessment (detection bias) | Low risk | Quote: “Care was taken to blind staff who performed anthropometric measurements at delivery.” |
| Incomplete outcome data (attrition bias) | Low risk | Comment: anthropometric measures at birth were available for 87% of the enrolled participants. |
| Selective reporting (reporting bias) | Low risk | Comment: all outcomes were presented in the paper. |
| Other bias | Low risk | Comment: no other bias was identified. |

**Kaestel^10^:**

| **Bias** | **Authors’ Judgement** | **Support for Judgement** |
| --- | --- | --- |
| Random sequence generation (selection bias) | Low risk | Quote: "Simple block randomisation with a block size of 150 was managed as follows: at entry, the project midwife randomly drew 1 piece of coloured paper corresponding to the colour code on the tablet containers from envelopes with initially 50 pieces of each of the three colours"  Comment: probably done. |
| Allocation concealment (selection bias) | Unclear risk | Quote: "at entry, the project midwife randomly drew one piece of coloured paper corresponding to the colour code on the tablet containers from envelopes with initially 50 pieces of each of the three colours"  Comment: insufficient evidence to determine whether allocation was concealed following generation of the randomisation sequence. |
| Blinding of participants and personnel (performance bias) | Low risk | Quote: "three identical-looking micronutrient supplements", "code was kept secret from study participants, study personnel, and data analysts until data cleaning and preliminary data analysis had been carried out." and "the health workers who collected outcome data after delivery did not have any knowledge of intervention group of the women" Comment: participants and caregivers were probably blinded to the treatment assignment. |
| Blinding of outcome assessment (detection bias) | Low risk | Quote: "three identical-looking micronutrient supplements", "code was kept secret from study participants, study personnel, and data analysts until data cleaning and preliminary data analysis had been carried out." and "the health workers who collected outcome data after delivery did not have any knowledge of intervention group of the women" Comment: outcome assessors were probably blinded to the treatment assignment. |
| Incomplete outcome data (attrition bias) | High risk | Exclusion (3.1%) and attrition (20.4%) data were reported along with their reasons. |
| Selective reporting (reporting bias) | Low risk | Comment: all outcomes mentioned in the methods section were presented in the paper. |
| Other bias | Low risk | Comment: no other bias was identified. |

**MINIMAt^11^:**

| **Bias** | **Authors’ Judgement** | **Support for Judgement** |
| --- | --- | --- |
| Random sequence generation (selection bias) | Unclear risk | Quote: "individual randomisation was done in blocks of 12" and "After enrolment, women were randomly assigned to 6 intervention groups".  Comment: method used for generating the randomisation sequence was not described in sufficient detail to permit judgement. |
| Allocation concealment (selection bias) | Unclear risk | Comment: method used for allocation concealment was not described in sufficient detail to permit judgement. |
| Blinding of participants and personnel (performance bias) | Low risk | Quote: "pills were identical in appearance, and monthly supplies were provided in identical bottles", " the mothers were unaware of their micronutrient supplement" and "double masking was practiced" Comment: study participants and caregivers were blinded to the treatment assignment. |
| Blinding of outcome assessment (detection bias) | Low risk | Quote: "pills were identical in appearance, and monthly supplies were provided in identical bottles", "the testers were unaware of children’s groups" and "double masking was practiced" Comment: outcome assessors were blinded to the treatment assignment. |
| Incomplete outcome data (attrition bias) | High risk | Attrition was (26%), reported along with their reasons. |
| Selective reporting (reporting bias) | Low risk | Comment: all outcomes mentioned in the methods section were presented in the paper. |
| Other bias | Low risk | Comment: no other bias was identified. |

**Osrin^12^:**

| **Bias** | **Authors’ Judgement** | **Support for Judgement** |
| --- | --- | --- |
| Random sequence generation (selection bias) | Low risk | Quote: "Randomly allocated 1200 participant identification numbers by computer into two groups in permuted blocks of 50".  Comment: probably done |
| Allocation concealment (selection bias) | Low risk | Quote: "We did randomisation in advance of recruitment", "The allocation code was kept on file in Kathmandu and London. We allocated every identification number a supplement container to last throughout the trial. Containers were filled with either intervention or control tablets in Kathmandu by a team member who was otherwise uninvolved in the trial; these containers were then marked only with identification numbers and transported to the study centre in Janakpur" and "After screening, consent, and enrolment, one of us (YS) allocated participants sequential identification numbers and the corresponding supplement containers".  Comment: probably done |
| Blinding of participants and personnel (performance bias) | Low risk | Quote: "The allocation code was kept on file in Kathmandu and London" and "Containers were filled with either intervention or control tablets in Kathmandu by a team member who was otherwise uninvolved in the trial; these containers were then marked only with identification numbers and transported to the study centre in Janakpur. Intervention and control supplements were manufactured to look, smell, and taste identical" Comment: participants and caregivers were probably blinded to the treatment assignment. |
| Blinding of outcome assessment (detection bias) | Low risk | Quote: "The allocation code was kept on file in Kathmandu and London" and "Containers were filled with either intervention or control tablets in Kathmandu by a team member who was otherwise uninvolved in the trial; these containers were then marked only with identification numbers and transported to the study centre in Janakpur. Intervention and control supplements were manufactured to look, smell, and taste identical" Comment: outcome assessors were probably blinded to the treatment assignment. |
| Incomplete outcome data (attrition bias) | High risk | Attrition was 5% and reasons for it were reported. Exclusion was 39.5% and reasons were not reported. |
| Selective reporting (reporting bias) | Low risk | Comment: all outcomes mentioned in the methods section were presented in the paper. |
| Other bias | Low risk | Comment: no other bias was identified. |

**Roberfroid^13^:**

| **Bias** | **Authors’ Judgement** | **Support for Judgement** |
| --- | --- | --- |
| Random sequence generation (selection bias) | Low risk | Quote: "The randomisation scheme was generated by a computer program in permuted blocks of 4".  Comment: probably done |
| Allocation concealment (selection bias) | Low risk | Quote: "Randomization numbers were sealed in opaque envelopes. At each inclusion, the consulting physician opened the next sealed envelope and transmitted the randomisation number to a pharmacist managing the allocation sequence and the packaging of drugs in Center Muraz. The pharmacist was also blinded to the intervention. Individual plastic zip bags contained 31 tablets each and were labelled with the participant’s name, address, and identification numbers only"  Comment: probably done |
| Blinding of participants and personnel (performance bias) | Low risk | Quote: "double blind", "Intervention and control micronutrient tablets were identical in appearance" and "code was kept secret from study participants and staff until completion of preliminary data analysis" and "Pharmacist was also blinded to the intervention". Comment: participants and caregivers were probably blinded to the treatment assignment. |
| Blinding of outcome assessment (detection bias) | Low risk | Quote: "double blind", "Intervention and control micronutrient tablets were identical in appearance" and "code was kept secret from study participants and staff until completion of preliminary data analysis" and "Pharmacist was also blinded to the intervention". Comment: outcome assessors were probably blinded to the treatment assignment. |
| Incomplete outcome data (attrition bias) | Low risk | Attrition was 7.5% and reason for it was provided. Only 1 woman was excluded because of therapeutic abortion. |
| Selective reporting (reporting bias) | Low risk | Comment: all outcomes mentioned in the methods section were presented in the paper. |
| Other bias | Low risk | Comment: no other bias was identified. |

**Shankar^14^:**

| **Bias** | **Authors’ Judgement** | **Support for Judgement** |
| --- | --- | --- |
| Random sequence generation (selection bias) | Low risk | Quote: “Before enrolment, midwife identification numbers were sequentially allocated to computer-generated, randomly permuted blocks of groups numbered one to eight, stratified by community health centre or village health clinic.” |
| Allocation concealment (selection bias) | Low risk | Quote: “All strips of supplements had an embossed batch number with an embedded digit designating one of eight groups, with four being IFA and the other four MMN. The code to indicate which strip was IFA or MMN was known only by the manufacturing production manager and a quality control officer from UNICEF, Copenhagen, neither of whom had any connection to the study or its personnel. The code was later transmitted in a sealed envelope from the production manager directly to the chairman of the data and safety monitoring board. All study scientists and personnel, government staff, and enrolees were unaware of the allocation.” |
| Blinding of participants and personnel (performance bias) | Low risk | See above (allocation concealment).  Quote: “Two types of identical-looking capsules, either IFA or MMN, were manufactured…” and “Warehouse staff selected designated supplement batches, relabelled them with the assigned midwife identification number in large black digits, and sent them to midwives. All pregnant women served by the same midwife received supplements with the same midwife identification number, and were of a clustered unit of randomisation.” |
| Blinding of outcome assessment (detection bias) | Low risk | See above (allocation concealment).  Comment: outcome assessment was done by maternal data collectors, midwives, or SUMMIT community facilitators who were probably blinded to allocation. |
| Incomplete outcome data (attrition bias) | Low risk | Quote: “Birth outcomes were known in 14 633 women in the IFA group and 14 909 women in the MMN group, yielding a 5·0% and 5·7% loss to follow-up during pregnancy and the loss of a one-woman cluster, with an additional loss from livebirth to 12 weeks post partum of 3·9% (n=553) in the IFA group and 4·0% (n=575) in the MMN group.” |
| Selective reporting (reporting bias) | Low risk | Comment: all outcomes presented in the methods were reported on. |
| Other bias | Low risk | Comment: no other bias was identified. |

**West (JiVitA-3)^15^:**

| **Bias** | **Authors’ Judgement** | **Support for Judgement** |
| --- | --- | --- |
| Random sequence generation (selection bias) | Low risk | Quote: "We used an in-house program (VBScript, Microsoft) that recognized 70 possible permutations for n=8 sectors and k=2 supplement allocations and 6 for the last block of n=4 sectors. Using this program, we randomized sectors within blocks to 1 of 2 codes such that each permutation had an equal probability of being chosen." |
| Allocation concealment (selection bias) | Low risk | Quote: "The resulting 2 lists of sectors were securely transmitted to field headquarters. One envelope with the code key was securely transmitted to the supplement producer and the other sealed in an envelope and secured at Johns Hopkins. At no time during the trial did study investigators or field or data management staff have access to the key." |
| Blinding of participants and personnel (performance bias) | Low risk | Quote: "double-masked", "Double Blind (Subject, Caregiver, Investigator, Outcomes Assessor)", "received daily supplementation, so treatment effect (still blinded due to the ongoing trial)"  Comment: probably done |
| Blinding of outcome assessment (detection bias) | Low risk | Quote: "double-masked", "Double Blind (Subject, Caregiver, Investigator, Outcomes Assessor)", "received daily supplementation, so treatment effect (still blinded due to the ongoing trial)"  Comment: probably done |
| Incomplete outcome data (attrition bias) | Low risk | Complete information was not available as the main trial has not been published; however, attrition is reported to be < 20% (trial presentations). |
| Selective reporting (reporting bias) | Low risk | Comment: reports from the study are still being published |
| Other bias | Low risk | Comment: no other bias was identified, including cluster-design specific biases (recruitment bias, baseline imbalance, loss of clusters, incorrect analysis, and comparability with individually randomised trials) |

**West (JiVitA-1)^16^:**

| **Bias** | **Authors’ Judgement** | **Support for Judgement** |
| --- | --- | --- |
| Random sequence generation (selection bias) | Low risk | Quote: “Sectors were randomized, blocked on every ninth, to achieve geographic and numerical balance using a traditional method whereby 9 coins of identical size (3 per code) were drawn in a blinded fashion and assigned to each sequential sector. The blocked drawing exercise was repeated 67 times until all 596 sectors were assigned codes representing placebo (n = 198), vitamin A (n = 198), or beta carotene (n=200).” |
| Allocation concealment (selection bias) | Low risk | Quote: “Prior to the trial, sectors were contiguously listed geographically, assigned a number from 001 to 596, and randomized to 1 of 3 codes (1, 2, or 3), each representing 1 of the 3 batches of supplements confidentially labeled by the supplement packer and shipper (Amway Nutrilite Health Institute, Buena Park, California).“ |
| Blinding of participants and personnel (performance bias) | Low risk | Quote: “Three batches of encapsulated oily supplements of identical size and color…” and “Prior to dispensing to the field, bottles were relabeled with 3-digit sector numbers to ensure correct delivery and to further mask teams.” |
| Blinding of outcome assessment (detection bias) | Low risk | See above (blinding of participants and personnel).  Quote: “..double-blind..”  Comment: probably done. |
| Incomplete outcome data (attrition bias) | Low risk | Quote: “A total of 60 294 identified pregnancies met the inclusion criteria (20 060 in the placebo group, 20 012 in the vitamin A group, and 20 222 in the beta carotene group). Of these, 59 666 (99% in each group) consented, had a known pregnancy outcome, and had vital status recorded at 12 weeks after pregnancy (19 862 in the placebo group, 19 806 in the vitamin A group, and 19 998 in the beta carotene group).” |
| Selective reporting (reporting bias) | Low risk | Comment: all outcomes were reported on |
| Other bias | Low risk | Comment: no other bias was identified |

**West (NNIPS-2)^17^:**

| **Bias** | **Authors’ Judgement** | **Support for Judgement** |
| --- | --- | --- |
| Random sequence generation (selection bias) | Low risk | Quote: “All wards were assigned in Kathmandu by a random draw of numbered chits, blocked on subdistrict, for eligible women to receive one of three identical coded supplements.” |
| Allocation concealment (selection bias) | Unclear risk | Comment: method for allocation concealment was not described in order to permit judgement. |
| Blinding of participants and personnel (performance bias) | Low risk | Quote: “These were opaque, gelatinous capsules containing peanut oil and 23 300 IU of preformed vitamin A as retinyl palmitate, 42 mg of all trans β carotene, or no vitamin A or β carotene (placebo).” |
| Blinding of outcome assessment (detection bias) | Low risk | See above (blinding of participants and personnel).  Quote: “..double-blind..” and “These data were reviewed and a “proximate” cause of death assigned by two doctors, one of whom was an obstetrician-gynaecologist; both were blind to treatment allocation.” |
| Incomplete outcome data (attrition bias) | Low risk | Quote: ”In all, 1136 (2.5%) women were excluded because they emigrated before becoming pregnant or dying or because they declined to be recruited.”  Comment: 43, 70, and 44 women lost to follow up by 12 weeks in placebo, vitamin A, and beta carotene groups, respectively. |
| Selective reporting (reporting bias) | Low risk | Comment: all outcomes were reported on. |
| Other bias | Low risk | Comment: no other bias was identified. |

**WHO^18^:**

| **Bias** | **Authors’ Judgement** | **Support for Judgement** |
| --- | --- | --- |
| Random sequence generation (selection bias) | Low risk | Quote: “Randomization lists for each site were produced with computer-generated random number blocking with randomly varying groups of 6 to 8 women and were used to restrict randomization within the strata (country).” |
| Allocation concealment (selection bias) | Low risk | Quote: “Randomization was performed independently for each study site by the Clinical Trials Unit at WHO/HRP Geneva, Switzerland, and transmitted only to the labeling and packaging company” and “The technique consisted of allocating consecutively numbered treatment boxes for each woman. Randomization codes remained at the WHO Clinical Trial Unit until the time of analysis and were not available to any person until the analyses were completed.” |
| Blinding of participants and personnel (performance bias) | Low risk | Quote: “The chewable tablets contained calcium carbonate or placebo. They were identical in form, color, and taste.” |
| Blinding of outcome assessment (detection bias) | Low risk | Quote: “Complete sets [of boxes and tablet bottles], all of which were identical, were shipped by WHO to centers and kept in a designated area with a locker. Individual treatment boxes were provided consecutively to the randomized subject.”  Comment: probably done |
| Incomplete outcome data (attrition bias) | Low risk | Quote: “Data from all subjects were included, irrespective of compliance or follow-up failures (intention-to-treat principle); women contributed with all available data until the time when they were lost to follow up.”  Comment: 3.4% of women in the calcium group and 3.7% of women in the placebo group lost to follow-up. |
| Selective reporting (reporting bias) | Low risk | Comment: all outcomes were reported on. |
| Other bias | Low risk | Comment: no other bias was identified. |

**Zagre^19^:**

| **Bias** | **Authors’ Judgement** | **Support for Judgement** |
| --- | --- | --- |
| Random sequence generation (selection bias) | Unclear risk | Quote: "Villages - not individuals were randomly assigned to one treatment group or the other"  Comment: method used for generating the randomisation sequence was not described in sufficient detail to permit judgement. |
| Allocation concealment (selection bias) | Unclear risk | Comment: method used for allocation concealment was not described to permit judgement. |
| Blinding of participants and personnel (performance bias) | Low risk | Quote: "Because the two supplements did not look identical and may have been recognizable, a coding system was put in place by the SONIPHAR pharmaceutical company in Niger. Six codes were assigned to the treatments: three for iron/folic acid and three for multimicronutrient supplements. SONIPHAR packaged the supplements in boxes with identical labelling except for the supplement code. Health workers, traditional midwives, and data collectors were informed that each supplement came in two sizes and colors, so that the code letter did not distinguish which supplement was used". Comment: participants and caregivers were probably blinded to the treatment assignment. |
| Blinding of outcome assessment (detection bias) | Low risk | Quote: "Because the two supplements did not look identical and may have been recognizable, a coding system was put in place by the SONIPHAR pharmaceutical company in Niger. six codes were assigned to the treatments: three for iron/folic acid and three for multimicronutrient supplements. SONIPHAR packaged the supplements in boxes with identical labelling except for the supplement code. Health workers, traditional midwives, and data collectors were informed that each supplement came in two sizes and colors, so that the code letter did not distinguish which supplement was used". Comment: outcome assessors were probably blinded to the treatment assignment. |
| Incomplete outcome data (attrition bias) | Unclear risk | Attrition was 18%. Reasons for attrition were reported, and dropout was significantly higher in the MMN (25/1893 (1.3%)) versus IFA (8/1777 (0.5%)) group. Exclusion data were not reported. |
| Selective reporting (reporting bias) | Low risk | Comment: all outcomes mentioned in the methods section were presented in the paper. |
| Other bias | Low risk | Comment: no other bias was identified, including cluster-design-specific biases (recruitment bias, baseline imbalance, loss of clusters, and comparability with individually randomised trials). Any incorrect analysis was corrected by adjustment for clustering within data reported in this review. |

**Zeng^20^:**

| **Bias** | **Authors’ Judgement** | **Support for Judgement** |
| --- | --- | --- |
| Random sequence generation (selection bias) | Low risk | Quote: "The randomisation schedule was generated off site with a pseudo-random number generator in SAS".  Comment: probably done |
| Allocation concealment (selection bias) | Low risk | Quote: "The randomisation schedule was generated off site with a pseudo-random number generator in SAS version 6 (SAS Institute, Cary, NC). A treatment colour code was assigned to each village based on the treatment allocation schedule".  Comment: probably done |
| Blinding of participants and personnel (performance bias) | Low risk | Quote: "double blind", "treatment colour code was assigned to each village based on the treatment allocation schedule. The treatment codes were opened only once all data had been collected and blinded analysis of the primary hypothesis was completed" and "were of identical appearance and packaged in blister packs"  Comment: participants and caregivers were blinded to the treatment assignment. |
| Blinding of outcome assessment (detection bias) | Low risk | Quote: "double blind", "treatment colour code was assigned to each village based on the treatment allocation schedule. The treatment codes were opened only once all data had been collected and blinded analysis of the primary hypothesis was completed"  Comment: outcome assessors were blinded to the treatment assignment. |
| Incomplete outcome data (attrition bias) | Low risk | Exclusion (4.8%) and attrition (2.3%) were reported along with their reasons |
| Selective reporting (reporting bias) | Low risk | Comment: all outcomes mentioned in the methods section were presented in the paper |
| Other bias | Low risk | Comment: no other bias was identified, including cluster-design-specific biases (recruitment bias, baseline imbalance, loss of clusters, and comparability with individually randomised trials). Investigators did not adjust for the cluster-randomised design in their sample size or outcome estimations, but this was corrected. |

References:

1) Adu-Afarwuah S, Lartey A, Okronipa H, et al. Lipid-based nutrient supplement increases the birth size of infants of primiparous women in Ghana. *Am J Clin Nutr.* 2015;101(4):835-846. doi: 10.3945/ajcn.114.091546.

2) Ashorn P, Alho L, Ashorn U, et al. The impact of lipid-based nutrient supplement provision to pregnant women on newborn size in rural Malawi: A randomized controlled trial. *Am J Clin Nutr.* 2015;101(2):387-397. doi: 10.3945/ajcn.114.088617.

3) Belizán JM, Villar J, Gonzalez L, et al. Calcium supplementation to prevent hypertensive disorders of pregnancy. *N Engl J Med.* 1991;325:1399-1405.

4) Bhutta ZA, Rizvi A, Raza F, et al. A comparative evaluation of multiple micronutrient and iron-folic acid supplementation during pregnancy in Pakistan: Impact on pregnancy outcomes. *Food Nutr Bull.* 2009;30(4 Suppl):S496-505. doi: 10.1177/15648265090304S404.

5) Christian P, Khatry SK, Katz J, et al. Effects of alternative maternal micronutrient supplements on low birth weight in rural Nepal: Double blind randomised community trial. *BMJ.* 2003;326(7389):571. doi: 10.1136/bmj.326.7389.571.

6) Mridha MK, Matias SL, Chaparro CM, et al. Lipid-based nutrient supplements for pregnant women reduce newborn stunting in a cluster-randomized controlled effectiveness trial in Bangladesh. *Am J Clin Nutr.* 2016;103(1):236-49.

7) Fawzi WW, Msamanga GI, Urassa W, et al. Vitamins and perinatal outcomes among HIV-negative women in Tanzania. *N Engl J Med.* 2007;356(14):1423-1431. doi: 10.1056/NEJMoa064868.

8) Friis H, Gomo E, Nyazema N, et al. Effect of multimicronutrient supplementation on gestational length and birth size: A randomized, placebo-controlled, double-blind effectiveness trial in Zimbabwe. *Am J Clin Nutr.* 2004;80(1):178-184. doi: 10.1093/ajcn/80.1.178.

9) Huybregts L, Roberfroid D, Lanou H, et al. Prenatal food supplementation fortified with multiple micronutrients increases birth length: a randomized controlled trial in rural Burkina Faso. *Am J Clin Nutr.* 2009;90:1593-600.

10) Kaestel P, Michaelsen KF, Aaby P, Friis H. Effects of prenatal multimicronutrient supplements on birth weight and perinatal mortality: A randomised, controlled trial in Guinea-Bissau. *Eur J Clin Nutr.* 2005;59(9):1081-1089. doi: 10.1038/sj.ejcn.1602215.

11) Persson LA, Arifeen S, Ekstrom EC, et al. Effects of prenatal micronutrient and early food supplementation on maternal hemoglobin, birth weight, and infant mortality among children in Bangladesh: The minimat randomized trial. *JAMA.* 2012;307(19):2050-2059. doi: 10.1001/jama.2012.4061.

12) Osrin D, Vaidya A, Shrestha Y, et al. Effects of antenatal multiple micronutrient supplementation on birthweight and gestational duration in Nepal: Double-blind, randomised controlled trial. *Lancet.* 2005;365(9463):955-962. doi: 10.1016/S0140-6736(05)71084-9.

13) Roberfroid D, Huybregts L, Lanou H, et al. Effects of maternal multiple micronutrient supplementation on fetal growth: A double-blind randomized controlled trial in rural Burkina Faso. *Am J Clin Nutr.* 2008;88(5):1330-1340. doi: 10.3945/ajcn.2008.26296.

14) Supplementation with Multiple Micronutrients Intervention Trial (SUMMIT) Study Group. Effect of maternal multiple micronutrient supplementaiton on fetal loss and infant death in Indonesia: a double-blind cluster-randomised trial. *Lancet*. 2008;371(9608215):215-27.

15) West KP, Jr., Shamim AA, Mehra S, et al. Effect of maternal multiple micronutrient vs iron-folic acid supplementation on infant mortality and adverse birth outcomes in rural Bangladesh: The JiVitA-3 randomized trial. *JAMA.* 2014;312(24):2649-2658. doi: 10.1001/jama.2014.16819.

16) West KP, Christian P, Labrique AB, et al. Effects of Vitamin A or Beta Carotene Supplementation on Pregnancy-Related Mortality and Infant Mortality in Rural Bangladesh: A Cluster Randomized Trial. *JAMA*. 2011;305(19):1986-1995.

17) West KP, Katz J, Khatry SK, et al. Double blind, cluster randomised trial of low dose supplementation with vitamin A or β carotene on mortality related to pregnancy in Nepal. *BMJ* 1999;318(7183):570-575.

18) Villar J, Abdel-Aleem H, Merialdi M, et al. World Health Organization randomized trial of calcium supplementation among low calcium intake pregnant women. *American Journal of Obstetrics and Gynecology.* 2006;194:639-49.

19) Zagre NM, Desplats G, Adou P, Mamadoultaibou A, Aguayo VM. Prenatal multiple micronutrient supplementation has greater impact on birthweight than supplementation with iron and folic acid: A cluster-randomized, double-blind, controlled programmatic study in rural Niger. *Food Nutr Bull.* 2007;28(3):317-327. doi: 10.1177/156482650702800308.

20) Zeng L, Dibley MJ, Cheng Y, et al. Impact of micronutrient supplementation during pregnancy on birth weight, duration of gestation, and perinatal mortality in rural western China: Double blind cluster randomised controlled trial. *BMJ.* 2008;337:a2001. doi: 10.1136/bmj.a2001.

**Appendix 3: The Global Young Women’s Nutrition Investigators Group Members**

*In alphabetical order by first name

| NAME | SURNAME | AFFILIATION |
| --- | --- | --- |
| Ana P | Bétran | World Health Organization, Switzerland |
| Brietta M | Oaks | University of Rhode Island, USA |
| David | Osrin | Institute for Global Health, University College London, UK |
| Ellen | Piwoz | The Bill and Melinda Gates Foundation, USA |
| Exnevia | Gomo | University of Zimbabwe, Zimbabwe |
| Faruk | Ahmed | Griffith University, Australia |
| Henrik | Friis | University of Copenhagen, Denmark |
| José | Belizán | UNC Chapel Hill, USA |
| Kathryn | Dewey | University of California, Davis, USA |
| Keith | West | Johns Hopkins Bloomberg School of Public Health, USA |
| Lieven | Huybregts | International Food Policy Research Institute, USA |
| Lingxia | Zeng | School of Public Health, Xi’an Jiaotong University Health Science Centre, China |
| Michael | Dibley | The University of Sydney, Australia |
| Noel | Zagre | UNICEF Regional Office for West and Central Africa |
| Parul | Christian | Johns Hopkins Bloomberg School of Public Health, USA |
| Patrick W | Kolsteren | Ghent University, Belgium |
| Pernille | Kaestel | University of Copenhagen, Denmark |
| Robert E | Black | Johns Hopkins Bloomberg School of Public Health, USA |
| Shams | El Arifeen | ICDDRB, Bangladesh |
| Ulla | Ashorn | Tampere University, Faculty of Medicine and Health Technology, Finland |
| Wafaie | Fawzi | Harvard T.H. Chan School of Public Health, USA |
